# Silane-crosslinked graphene oxide reinforced chitosan/sodium alginate hydrogel for controlled release of insulin

**DOI:** 10.1039/d5ra02008e

**Published:** 2025-06-12

**Authors:** Faiza Zainab, Sadullah Mir, Sher Wali Khan, Nasser S. Awwad, Hala A. Ibrahium

**Affiliations:** a Department of Chemistry, COMSATS University Islamabad Islamabad Campus Pakistan sadullahmir@comsats.edu.pk +9203365270913; b Department of Chemistry, Rawalpindi Women University Rawalpindi Pakistan; c Chemistry Department, King Khalid University AlQura'a, Abha, P.O. Box 960 Saudi Arabia; d Biology Department, King Khalid University AlQura'a, Abha, P.O. Box 960 Saudi Arabia

## Abstract

Diabetic patients require regular insulin administration to maintain standard glucose. However, conventional injection procedures frequently lead to low patient compliance and an elevated risk of hypoglycemia. To overcome these challenges, a hydrogel-based system has been developed, with preliminary findings suggesting its potential for sustained insulin release while exhibiting pH-responsiveness and biocompatibility. A hydrogel composed of chitosan, sodium alginate, and graphene oxide (GO) crosslinked with tetra ethoxy silane (TEOS) was synthesized *via* the solvent casting method. The hydrogel's structural and physicochemical properties were determined by various analytical techniques. Swelling behavior, gel fraction, antimicrobial studies, insulin encapsulation efficacy, and *in vitro* release were analyzed at different pH. The hydrogel demonstrated higher encapsulation efficacy of 94% and pH-responsive swelling behavior with maximum swelling of 100% at pH 7. This hydrogel exhibited complete crosslinking, ensuring robust structural integrity and prolonged drug release as indicated by gel fraction analysis (almost 96–100%). Initially, *in vitro* studies were carried out to evaluate the insulin release profile. This study indicated that insulin release is pH-dependent, and it is a maximum of almost 98% at pH 6.8. SEM revealed the well-developed porous and spherical morphology. However, TGA and DSC confirmed its excellent thermal stability. Additionally, CSGI hydrogel exhibited a strong antimicrobial effect.

## Abstract 2

Synthesis, characterization, pharmacological evaluation, and antibacterial activity of silane-crosslinked graphene oxide reinforced chitosan/sodium alginate hydrogel for controlled insulin release.

## Graphical abstract

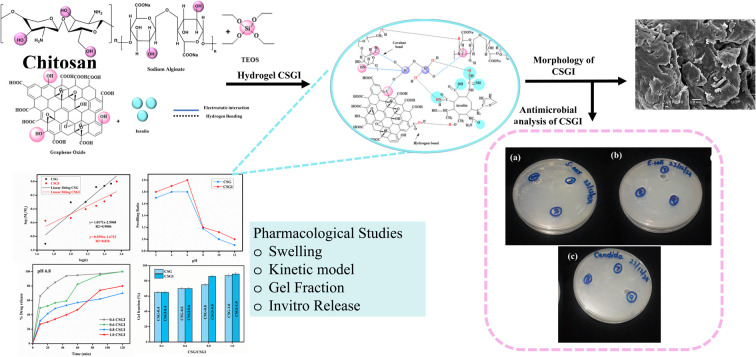

## 1. Introduction

One of the most prevalent diseases in the world today is diabetes mellitus. It is a growing global health concern, with cases that rose from 531 million in 2021 to 634 million by 2030, according to the International Diabetes Federation (IDF).^1^ Diabetes mellitus is a long-term chronic disorder resulting from elevated blood glycemic levels due to impaired insulin function. Diabetes is classified into two types: type 1 diabetes and type 2 diabetes. Type 1 diabetes occurs when β-cell function is completely lost, necessitating insulin therapy for survival. In contrast, type 2 diabetes is related to an increased risk of both vascular and non-vascular complications, including cardiovascular disease, cancer, infections, liver disease, and nervous system impairments.^2–5^ It requires a more tailored insulin therapy approach incorporating basal, mealtime, and correctional insulin strategies to manage this condition effectively.^6,7^

The traditional method of administering exogenous insulin involves syringes and insulin pens, which can lead to distress and trauma due to needle-induced pain. In this regard, biopolymeric hydrogels evolved into a leading alternative to insulin therapy, offering transformative potential in the drug delivery system.^8,9^ Hydrogels have a dimensional network, crosslinked by polymers that exhibit unique properties, making them suitable for biomedical applications and highly effective for insulin therapy.^10–13^ Biopolymeric hydrogels, derived from natural polymers, including chitosan and sodium alginate, offer significant advantages over conventional insulin delivery methods.^14^

Chitosan (CS) is a naturally occurring cationic polymer produced by the deacetylation of chitin. It contains glucosamine units and *N*-acetylglucosamine units interconnected by 1–4 glycosidic bonds. However, its mechanical strength is weak due to the presence of amine and hydroxyl groups.^15–17^ Due to this limitation, it is cross-linked with another natural polymer, sodium alginate, which is an anionic moiety. It is extracted from brown seaweed and formed from β-*d*-mannuronic acid (M) and α-*l*-guluronic acid (G) units that are connected by a glycosidic bond that defines its structural properties. Its carboxylic functional group contributes to its stabilizing, biocompatible, and pH-sensitive characteristics, making it highly versatile for biomedical applications.^18–21^

Graphene oxide (GO) is a sp^2^ hybridized carbon atom and serves as a promising candidate for enhancing the mechanical durability and swelling behavior of polymer-based hydrogels. Moreover, its exceptional properties, like electrostatic interaction, larger surface area, hydrogen bonding capabilities, and excellent dispersibility in water, have garnered significant interest for application in drug delivery.^22–24^ Wang Yu, *et al.*, synthesized a microneedle patch by modifying chitosan and alginate with phenylboronic acid and polyvinyl alcohol for insulin administration.^8^ Phan *et al.* fabricated core–shell hydrogel beads through a simple dropping method. To prevent insulin leakage, these hydrogels are coated with a double-layered hydroxide of chitosan and alginate to form hydrogel beads. To check its biocompatibility, the experiment was performed using a chick embryo model, and the insulin release rate in intestinal fluid was studied at pH 6.8. The release rate of insulin is more effective in harsh acidic conditions than in basic conditions.^25^

In this study, we aim to synthesize stable hydrogel CSGI to demonstrate its potential antidiabetic properties in an *in vitro* mode. Recent findings of chitosan, sodium alginate, and graphene oxide crosslinked with TEOS loaded with insulin-blended hydrogels have improved properties, including biocompatibility, cytotoxicity, and controlled release kinetics. The above cross-linked hydrogel locks insulin in this matrix and has % excellent encapsulation efficacy of 94%. Controlled drug release of insulin by hydrogels offers potential advantages of reduced injection frequency and enhanced patient compliance. However, further clinical validation, comprehensive safety assessment, and demonstrable therapeutic efficacy are essential to establish its potential as a viable approach for diabetes management.

## 2. Material and methods

### 2.1. Materials

Chitosan (0.15–0.3 g cm^−3^), sodium alginate, acetic acid, calcium chloride, sodium chloride TEOS, hydrochloric acid (HCl), ethanol, and NaOH were purchased from Sigma-Aldrich, USA. Graphene oxide was prepared using the Hummers' method. The insulin was provided by Feroze Sons, Pakistan.

### 2.2. Synthesis of graphene oxide

The Hummer's method was used for the synthesis of GO. In a 1-liter (1000-milliliter) volumetric flask, 2 g of graphite powder and 2 g of NaNO_3_ were combined with 50 ml of 98% H_2_SO_4_. For two hours, this solution was continuously stirred in an ice bath at 0–5 °C. To maintain the reaction's temperature below 15 °C, KMnO_4_ (6 g) was very slowly and carefully added to the suspension after two hours. The solution was stirred at 35 °C without the use of an ice bath until a pasty, greenish-black mixture was formed. For two days, there was more stirring. After two days, 100 ml of water was added to the solution very slowly, drop by drop. This raised the temperature of reaction to around 98 °C, at which point a brown froth became apparent. To dilute the mixture once again, 200 ml of water was added to the above solution and stirred constantly. To finish this reaction, 10 ml of H_2_O_2_ was added as the last treatment. The appearance of yellow served as confirmation that the termination had occurred. The combination was further refined by centrifuging it ten times in deionized water and washing it ten times in 10% HCl. After filtering and vacuum-drying at 90 °C, the GO was produced as a fine black nanosheet.^26^

### 2.3. Synthesis of sodium alginate

To prepare sodium alginate from alginic acid. The first step is to weigh 3.6 g of alginic acid, dissolve it in 300 ml of distilled water, and stir it for half an hour. Then, slowly add 1.5 g of NaOH (10–15% of alginic acid weight) by monitoring the pH to reach 7–8. This reaction will be carried out for an hour at room temperature. Then, filter the solution to remove any undissolved particles. Centrifugation will be carried out to collect the sodium alginate, which could be separated from the water. Then, it will be oven-dried at a temperature of 50–60 °C for 12 h. Now, the dried sodium alginate powder will be collected and used for hydrogel synthesis.^27,28^

### 2.4. Synthesis of hydrogel

**Fig. 1:**
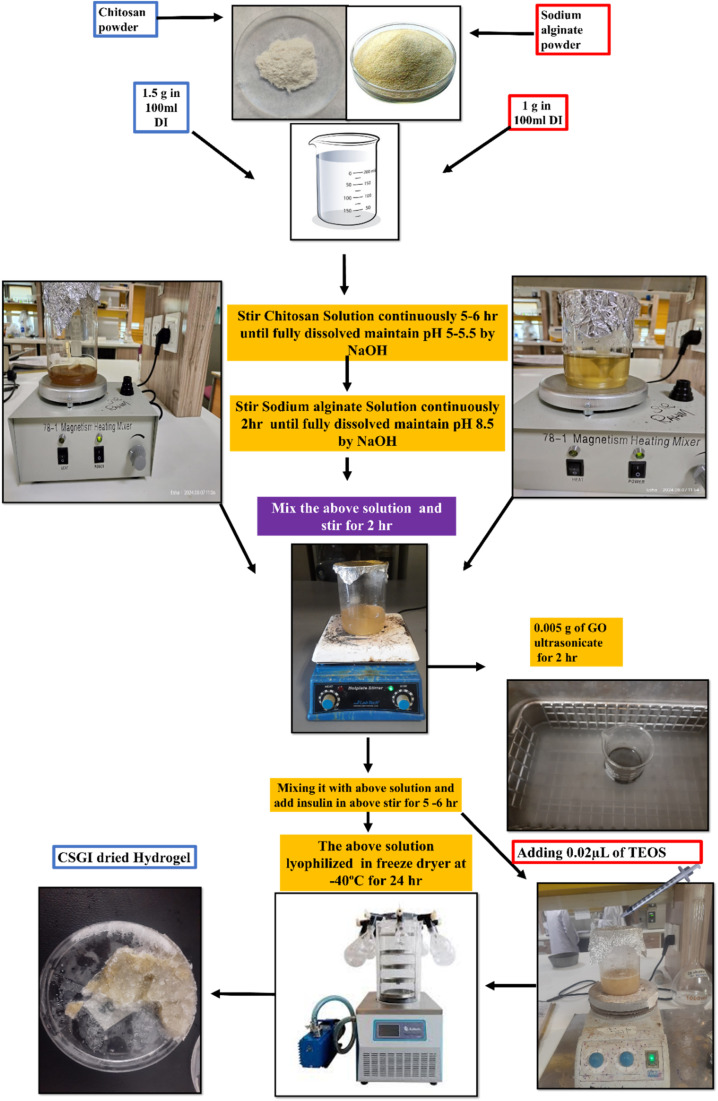
Synthesis of CSGI hydrogel.

First, weigh 1.5 g of chitosan and dissolve it in 100 ml of deionized water that contains 1% of acetic acid solution. Stir it continuously for 5–6 h until chitosan is fully dissolved to form a homogenous solution. Adjust the pH to 5–5.7 of the solution by using 1 M NaOH. Then separately add 1 g of sodium alginate in 50 ml of deionized water and stir it continuously for 2 h. Adjust the pH 8.5 of the solution by using 1 M NaOH while stirring. Mixing the above chitosan and sodium alginate solution continuously for 2 h to avoid lump formation to ensure complete blending. Then, separately weigh 0.005 g of graphene oxide, add 10 ml of DI, sonicate it for 2 h, and then stir it for half an hour to ensure uniform mixing. Then, add this to the above chitosan and sodium alginate solution. Then, add 100 units of insulin solution to the above mixture, stir it for 5–6 h, and ensure the temperature of the solution is kept at 4 °C by using an ice bath. Then, add 0.1 ml of TEOS in 10 ml of ethanol followed by the addition of HCL, and add this to the above solution dropwise while continuously stirring to crosslink chitosan sodium alginate, GO, and insulin solution. The prepared solution was added in a Petri dish and lyophilized at −40 °C and then After freeze-drying, the dried hydrogel (CSGI) is kept in a refrigerator to avoid denaturation, as shown in Fig. 1. We made concentrations of the above hydrogel without insulin similar to the above, and oven-dried at 60 °C for 24 h. This hydrogel (CSG) was used to determine various pharmaceutical studies.^25^

### 2.5. FTIR spectroscopy analysis

FTIR spectroscopy was used to study the possible intermolecular interactions in a hydrogel. Thermo Scientific Nicolet 6700 FTIR Spectrometer was used for the analysis. The samples of hydrogel were finely pulverized to create a pellet and combined with potassium bromide (KBr). To identify the vibrational modes of the functional groups and evaluate the chemical interactions inside the hydrogel matrix, the FTIR spectra were acquired over a range of 400 cm^−1^ to 4000 cm^−1^.^29^

### 2.6. XRD analysis

XRD analysis was used to identify the phase composition, crystal structure, and orientation of the above hydrogel samples. By using a PW3050/60 Goniometer (Theta/Theta) with Cu Kα radiation, the XRD patterns were obtained throughout a specified 2*θ* range of 5–79°. The device was running at 40 kV, 30 mA, and 1° min^−1^ scanning speed. The hydrogel samples' various characteristics, including crystallinity and structural orientation, were determined.^30^

### 2.7. DSC analysis

DSC analysis was used to study the thermal behavior of hydrogel CSGI to analyze its glass transition temperature (*T*_g_), melting points (*T*_m_), and decomposition temperature (*T*_d_) to assess drug–polymer interaction. Also, these results confirm the thermal stability and compatibility of the sample. The hydrogel sample was heated under a nitrogen atmosphere in an aluminum pan at 20° to 600 °C.^31^

### 2.8. TGA analysis

Thermal gravimetric analysis was utilized for the hydrogel using the Shimadzu DTG-60 to assess its thermal stability. The sample films of about 5 mg were deposited in an alumina pan with a nitrogen environment and a flow rate of 20 ml min^−1^. A temperature range of 20 to 600 °C was maintained. A rate of 10 °C per minute was used for scanning. An analysis and record were made of temperature variations and recurrent weight loss.^32^

### 2.9. SEM analysis

SEM was employed to look at the surface morphology of the hydrogel. And 5 kV to 15 kV was the operating voltage range for the Jeol JSM 6400 microscope. The samples were sputter-coated with a tiny layer of gold to enhance picture clarity and conductivity, and they were adhered to aluminum holders using sticky carbon tape. The hydrogel films' surface texture, porosity, homogeneity, and microstructural properties were all thoroughly examined by SEM analysis and are critical to any future uses.^33^

### 2.10. EDX analysis

EDX analysis was also used to assess the element composition of the alginate–chitosan hydrogel samples and the purity of the mixing ingredients. A Jeol, JSM 6400 scanning electron microscope was used to conduct the EDX examination. The voltage at which the instrument was operated ranged from 5 kV to 15 kV.^34^

### 2.11. Assessment of antimicrobial activities

The antimicrobial activity was assessed using the agar well diffusion method. The nutrient agar media was prepared and sterilized through autoclaving. This study cultured the bacterial and fungal strains of *Escherichia coli*, *Staphylococcus aureus*, and *Candida albicans* on nutrient agar media. In a Petri dish, 20 ml of media was poured and incubated at 37 °C for 2 h for stabilization. In a Petri dish, 10 mg of the CSGI sample of the plate is placed in a separate mark on the points. These plates were kept in a refrigerator at 4 °C for 3 h to allow diffusion and then incubated at 37 °C for 48 h. The zone of incubation was determined after 24 h and then after 48 h to check the extent of bacterial inhibition by the sample.^35–38^

### 2.12. Swelling studies of hydrogel

Hydrogel samples of CSG and CSGI swelling investigations were carried out in pH 2–12. Weigh out 0.01 g of each hydrogel sample precisely, then put them on Petri dishes with 30 ml of each buffer solution. Take out the hydrogels from the buffers after 50, 100, 150, 200, 250, 300, and 350 minutes, as determined by the time intervals. Also, by using different molar concentrations of NaCl and CaCl_2,_ its swelling was investigated. After using tissue paper to gently blot the samples to remove any excess buffer, weigh the samples with an analytical balance. To ensure dependability, the experiment was conducted three times. Using the eqn (1), the swelling ratio (SR) and water content (%) of the sample were determined by eqn (2):1
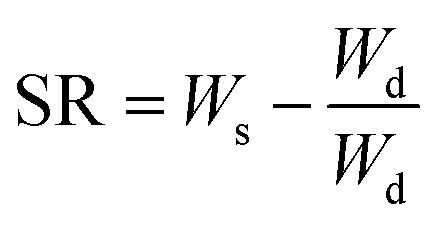
2
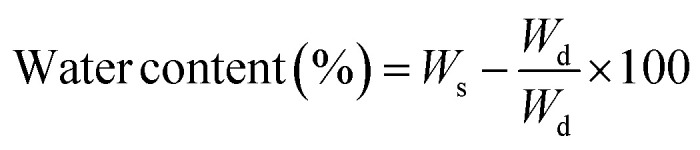
where *W*_s_ and *W*_d_ are the weights of the swollen and dried hydrogel samples.^39–42^

### 2.13. Gel fraction analysis

Gel fraction % is the total amount of non-dissolvable hydrogel indicated in the degree of crosslinking in insulin-loaded and unloaded hydrogel CSGI and CSG whose weight was indicated as (*W*_0_). The dried CSGI and CSG samples are immersed in distilled water for 8 h and magnetically stirred to remove soluble fractions, undissolved polymers, and free insulin. The swollen hydrogel samples were oven-dried at 40–45 °C, and their weight was measured later on as (*W*_g_)^43–45^ as shown in eqn (3).3
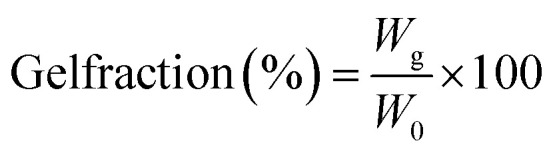


### 2.14. Erosion studies

Erosion studies were carried out after swelling studies. Every swollen sample was dried in a 40 °C oven for 12 hours, and until consistent mass was reached, each sample was separately weighed. There were two batches in which similar studies were carried out. The hydrogel erosion (%) is calculated at various time intervals by eqn (4).4
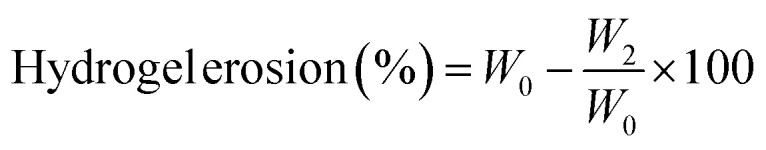
where *W*_0_ is the weight of the swollen wet hydrogel and *W*_2_ is the dried hydrogel final sample weight.^46^

### 2.15. Encapsulation efficacy

The encapsulation efficiency was determined by the method discussed in the literature. The sample CSGI of 20 mg was placed in 10 ml PBS buffer of pH = 7.4 in a shaking incubator for 24 h. After that, it was centrifuged. A UV-visible spectrophotometer determines the concentration of the supernatant. The encapsulation efficiency was determined by the following eqn (5):

5 Encapsulation efficiency (%) = total drug − free drug/total drug × 100

### 2.16. Post-encapsulation insulin stability assay

The stability of insulin after encapsulation is crucial to ensure that the hydrogel not only controls the release of insulin but also preserves its structural integrity. Protein-based drugs are prone to denaturation, especially insulin under physiological and storage conditions. Hence post post-encapsulation stability of insulin was determined to check its stability. In the present study, the insulin-loaded CSGI hydrogel was stored at 4 °C and sampled at regular intervals over 28 days. The release of insulin was quantified by using UV spectroscopy at 276 nm. The concentration of insulin at each point was calculated by a standard curve and expressed as % retained insulin against time.^47^

### 2.17. *In vitro* drug release studies

A dissolution study was performed for the *in vitro* release of a drug. Hydrogel was suspended in dissolution media at pH 1.2, 6.8, and 7.4, and continued stirring. We prepared the solution at pH 1.2 by using an HCl buffer. We continued stirring for 2 h, and for the solution at pH 6.8, we added phosphate buffer and continued stirring for 3 h. Continue stirring for 7 h by replacing it with fresh phosphate buffer media with pH 7.4. The whole study was performed at 37 °C, and samples were analyzed by UV-visible spectrophotometer.^48,49^

## 3. Results and discussion

### 3.1. Crosslinking mechanism of hydrogel

**Scheme 1:**
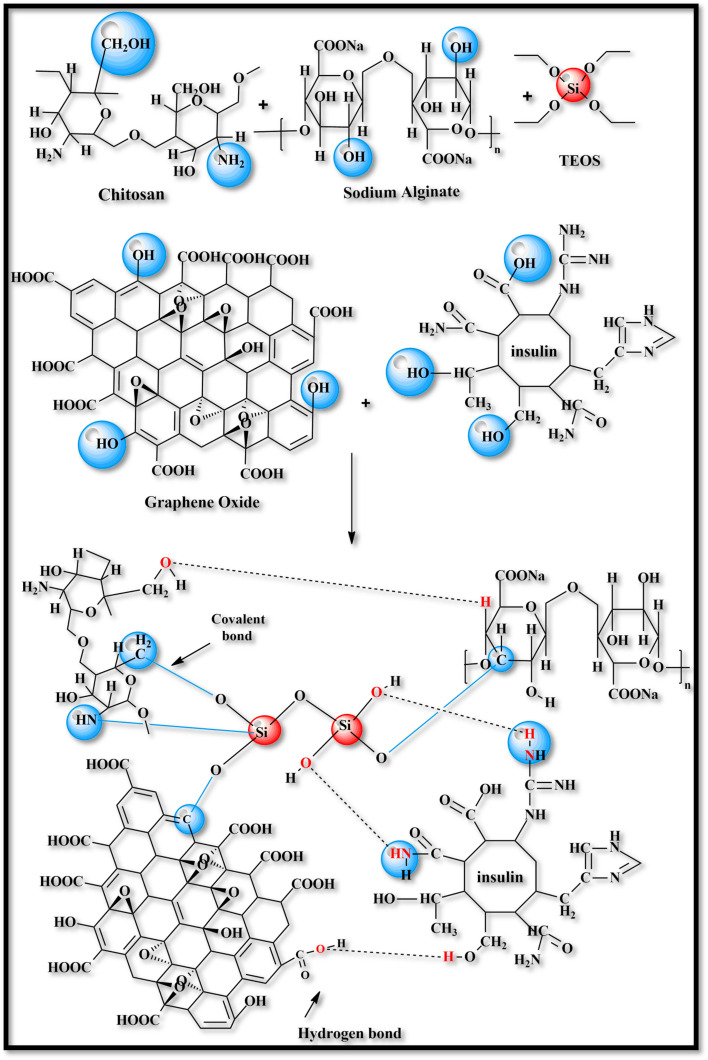
Crosslinking scheme of CSGI hydrogel.

The crosslinking mechanism of chitosan, sodium alginate graphene oxide, and tetraethyl orthosilicate involves hydrolysis and condensation of TEOS involving siloxane (Si–O–Si) bonds and silanol (Si–OH) through covalent interaction and hydrogen bonding, as shown in Scheme 1.^50,51^ Covalent interaction occurs through the amino group (–NH_2_) and hydroxyl group (–OH) of chitosan, and in sodium alginate, it forms stable bonds with the carboxyl group (–COOH) and hydroxyl groups (–OH) with silanol group (Si–OH). Graphene oxide interacts with its functional groups like carboxyl (–COOH), hydroxyl (–OH), and epoxy group with TEOS and polymer to enhance its mechanical strength and stability. Insulin interacts with it through the electrostatic interaction of amino acid groups, including amine group and carboxyl groups and hydrogen bonding. These interactions are capable of controlled and pH-responsive insulin release due to the hydrogel's porous and hydrophilic nature.^31,52^

### 3.2. FTIR analysis

**Fig. 2:**
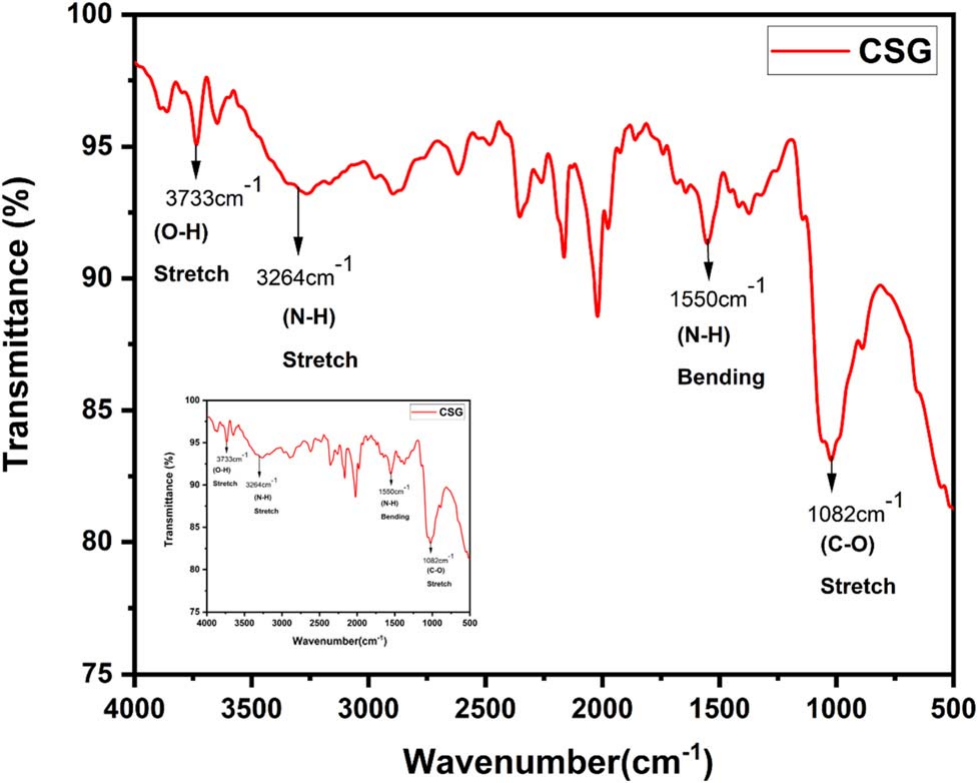
FTIR graph of pure chitosan.

**Fig. 3:**
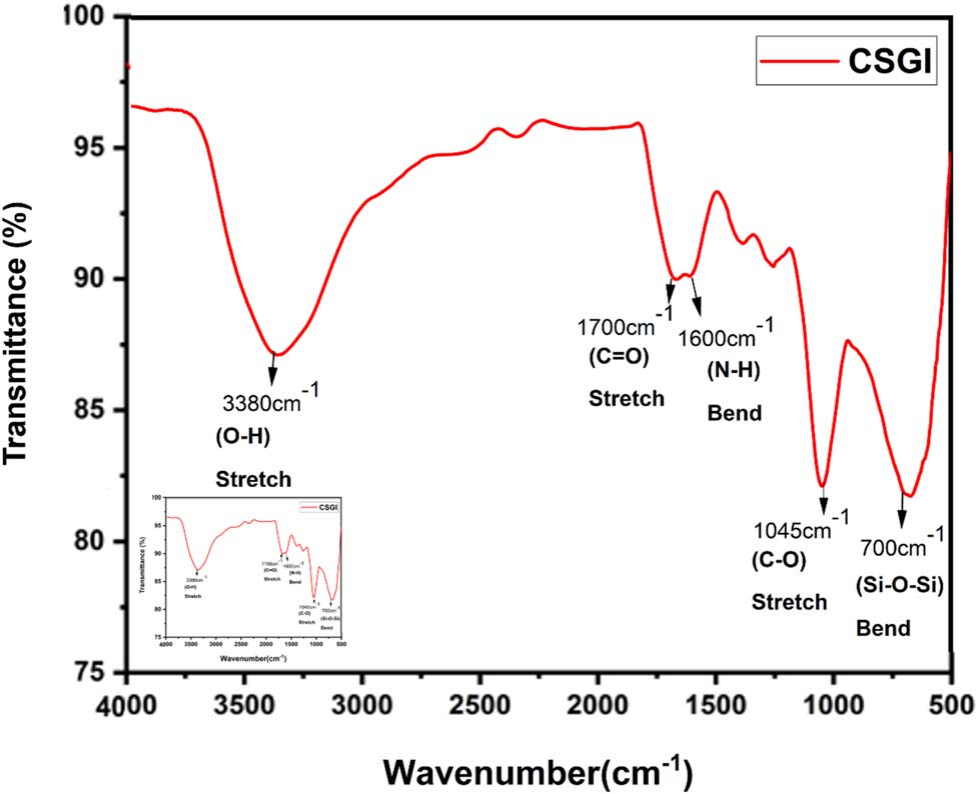
FTIR graph of CSGI hydrogel.

FTIR spectroscopy analysis of chitosan (CS) and CSGI hydrogel is shown in Fig. 2 and 3. The characteristic broader peak between 3400–3300 cm^−1^ is due to the stretching vibration of –NH and –OH groups overlapping, indicating the presence of chitosan. In addition, the peak is around 1650 cm^−1^ due to the C=O stretching vibration of the amide band I, which is the backbone of chitosan. In another amide band II, the peak is around 1550 cm^−1^, confirming the presence of chitosan from N–H bending and C–N stretching. The peaks around 1600–1400 cm^−1^ are due to the asymmetric and symmetric stretching of carboxylate groups, indicating the characteristics of sodium alginate^23,53,54^ The characteristic peak around 1720 cm^−1^ is due to the C=O stretching of the carboxyl group, confirming the presence of graphene oxide. The amide bands I and II represented the proteinaceous nature of insulin. The peaks around 1100–950 cm^−1^ are due to Si–O–Si and Si–OH stretching vibration, which explains the crosslinking mechanism by siloxane and silanol groups.^55–57^

### 3.3. XRD analysis

**Fig. 4:**
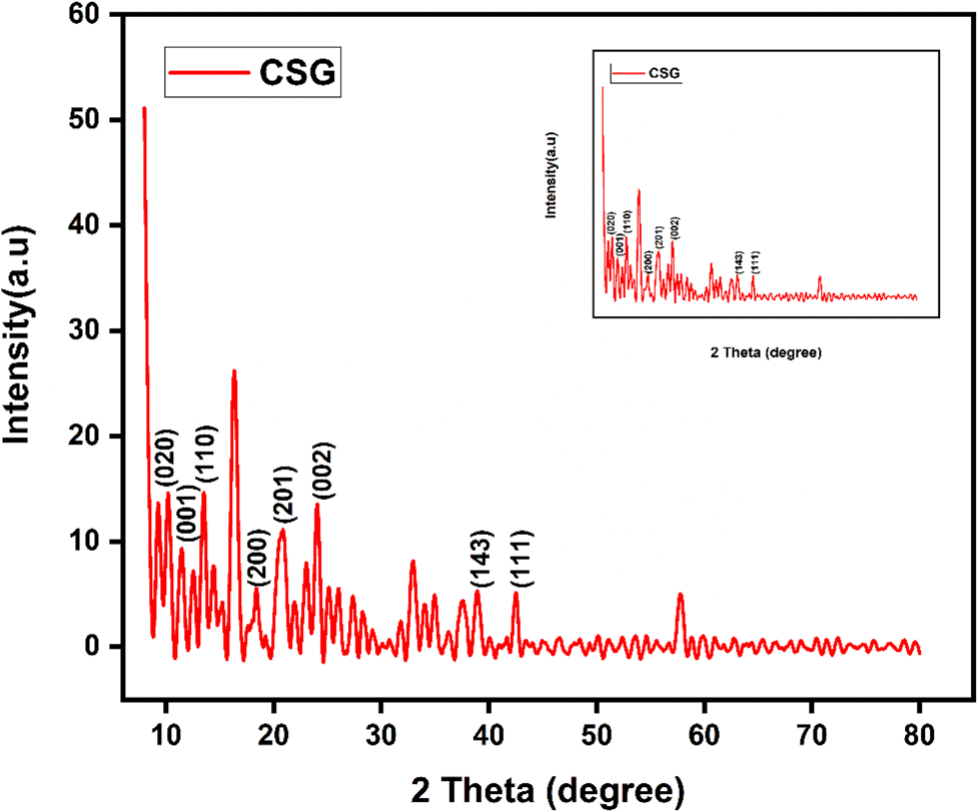
XRD graph of CSG hydrogel.

**Fig. 5:**
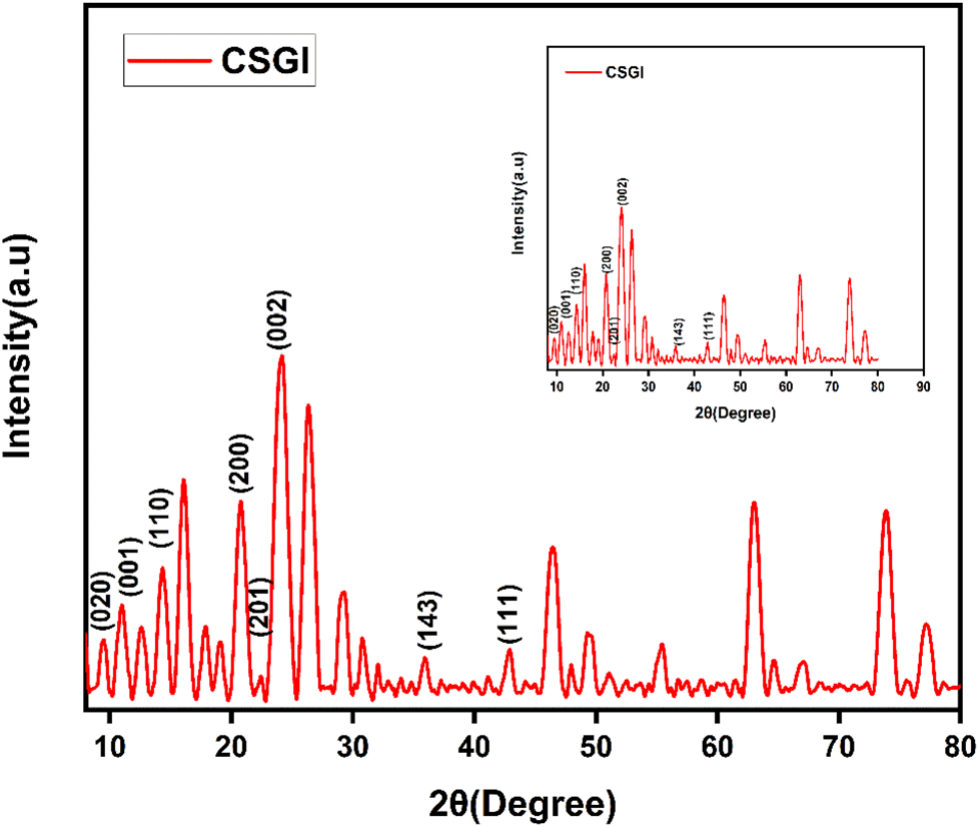
XRD graph of CSGI hydrogel.

The XRD analysis of chitosan, sodium alginate, and graphene oxide crosslinked by TEOS and loaded without insulin CSG and with insulin CSGI demonstrates the distinct structural features consistent with the literature, as shown in Fig. 4 and 5. Chitosan contributes characteristic peaks at 2*θ* at 10°, 2*θ* = 9.6°, 20°, 21.3°, and 38.7°, indicating its semicrystalline nature corresponding to (020), (200), (201), and (143). Sodium alginate exhibited 2*θ* = 13.6° and 21.5°, corresponding to the (110), (200) plane 2*θ* = 39° and indicating an amorphous structure. The incorporation of graphene oxide introduces a peak at 2*θ* = 10.6°, a plane (001), 2*θ* = 23.6° at (002); in the case of CSGI, it is at 24.3° and 26.6° prominent peak, and 2*θ* = 43.6° corresponding to the and (111) plane and reflects its layered structure.^58^ These features collectively indicate crystallinity and a semi-amorphous hydrogel network due to stronger interaction of CS, SA, and GO crosslinked by TEOS.^45,59–61^

### 3.4. DSC analysis

**Fig. 6:**
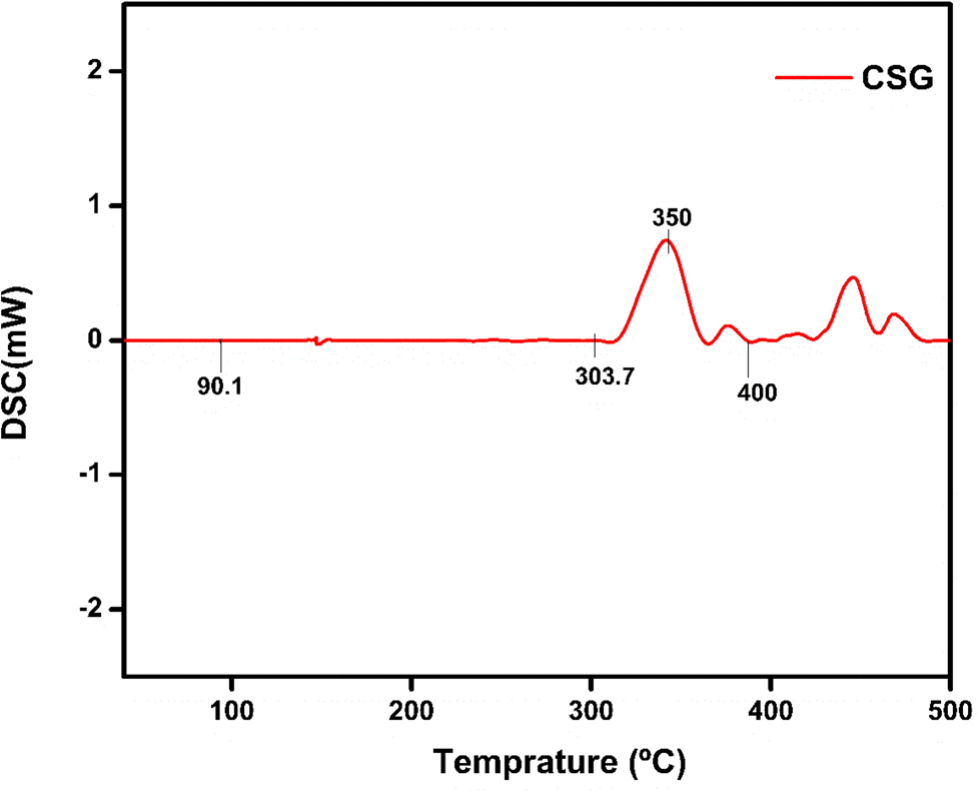
DSC Analysis CSG without insulin at a temperature range of 50 to 500 °C.

**Fig. 7:**
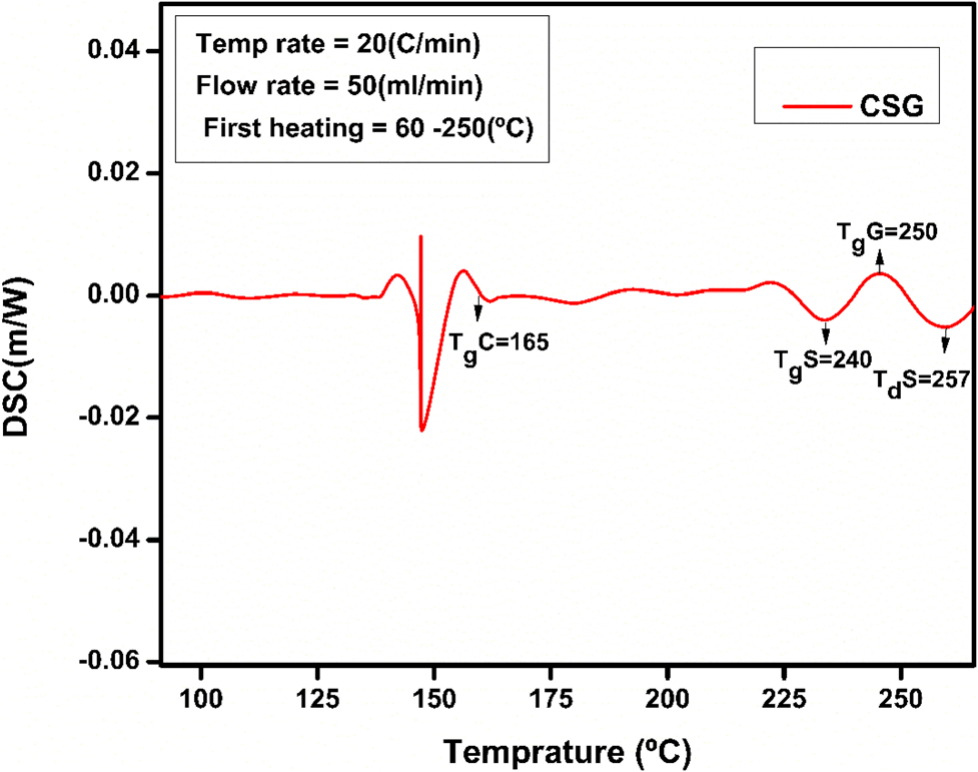
DSC of CSG representing peaks for chitosan and sodium alginate at a temperature range of 50 to 250 °C.

**Fig. 8:**
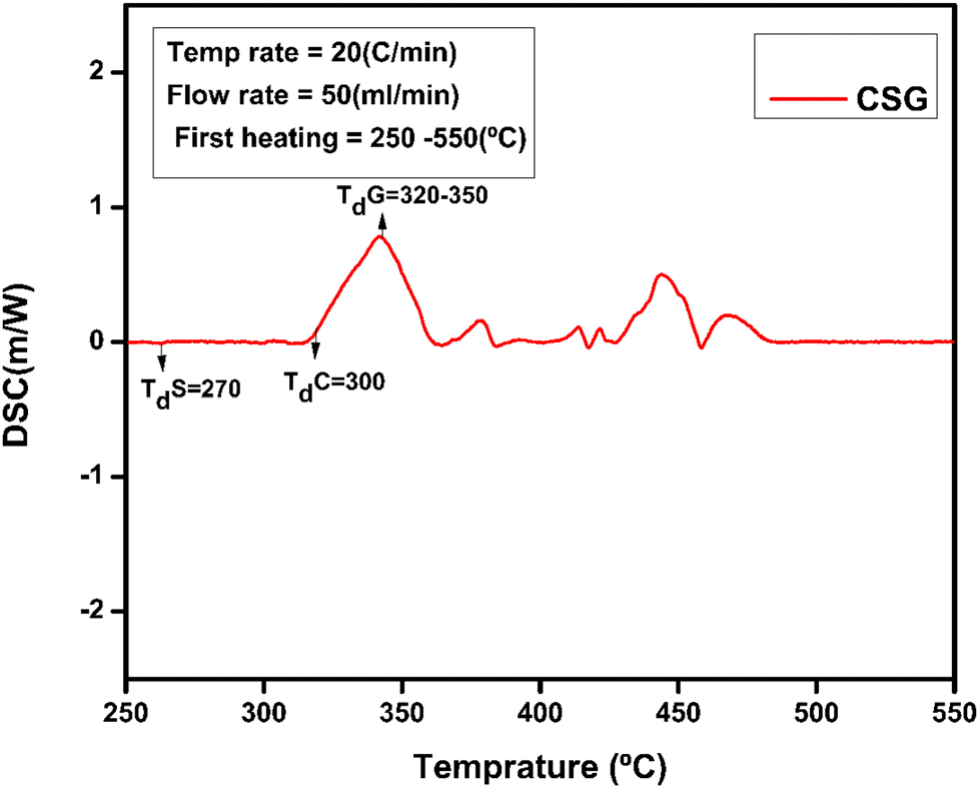
DSC of CSG hydrogel represents the chitosan, sodium alginate, and GO peaks.

The thermal and structural properties of hydrogel CSG hydrogel are represented in Fig. 6. The broader endothermic peak of chitosan is at 90 °C representing a loss of water and 165 °C attributing its glass-transition temperature also *T*_d_ the degradation temperature of polymer chitosan is at 300 °C which is an exothermic change due to the decomposition of amine group as shown in Fig. 7 and 8 of CSG^62,63^ The exothermic peak of *T*_g_ = 20 °C of sodium alginate also decomposition peak in a range of 257 °C depending on the heating rate.^31^ Also, graphene oxide has a peak around 200–250 °C representing transition and degradation.^64^

### 3.5. TGA analysis

**Fig. 9:**
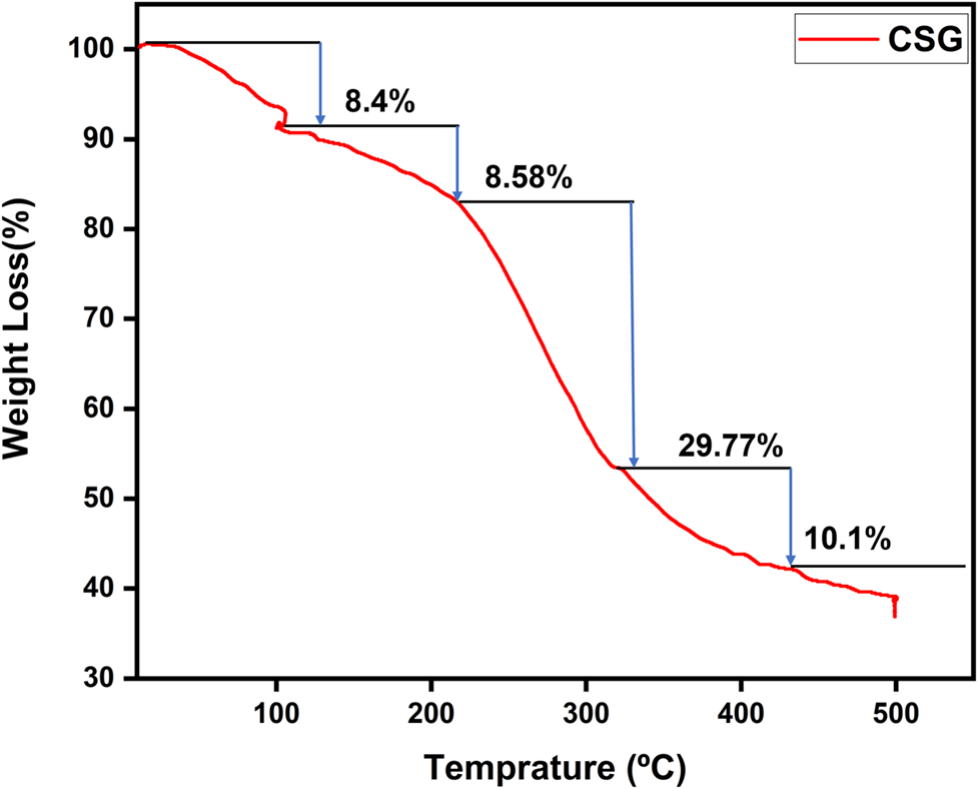
TGA analysis of CSG hydrogel.

TGA curves of compounds evaluate the weight variation and decomposition of rate of a material under an air atmosphere from room temperature to 1000 °C. TGA serves as an effective method for analyzing thermal stability and the decomposition behavior of polymers. The thermogravimetric analysis graph of hydrogel (CSG) shows the percentage of weight loss with temperature variation. The hydrogel CSG was heated from 100 °C to 500 °C. The weight loss occurs in four distinct stages: the first stage shows a weight loss of 8.4% up to around 106 °C, likely due to water loss.^30^ The second stage, with an 8.58% weight loss between approximately 200 °C and 300 °C, is indicative of the decomposition of volatile components. The third stage shows a 29.77.4% weight loss around 300 °C to 400 °C, suggesting further degradation of the chitosan and sodium alginate, possibly due to the breakdown of more stable organic fractions or the decomposition of residual materials.^31^ The final stage shows a weight loss of 10.1% up to 600 °C, indicating the decomposition of the Graphene oxide components or inorganic residues. These results indicate that the prepared hydrogel showed higher thermal stability compared to the pure chitosan in different temperatures, complex formation increased the thermal stability comparatively that is why more reside remain as shown in the Fig. 9.

### 3.6. Scanning electron microscopic analysis

**Fig. 10:**
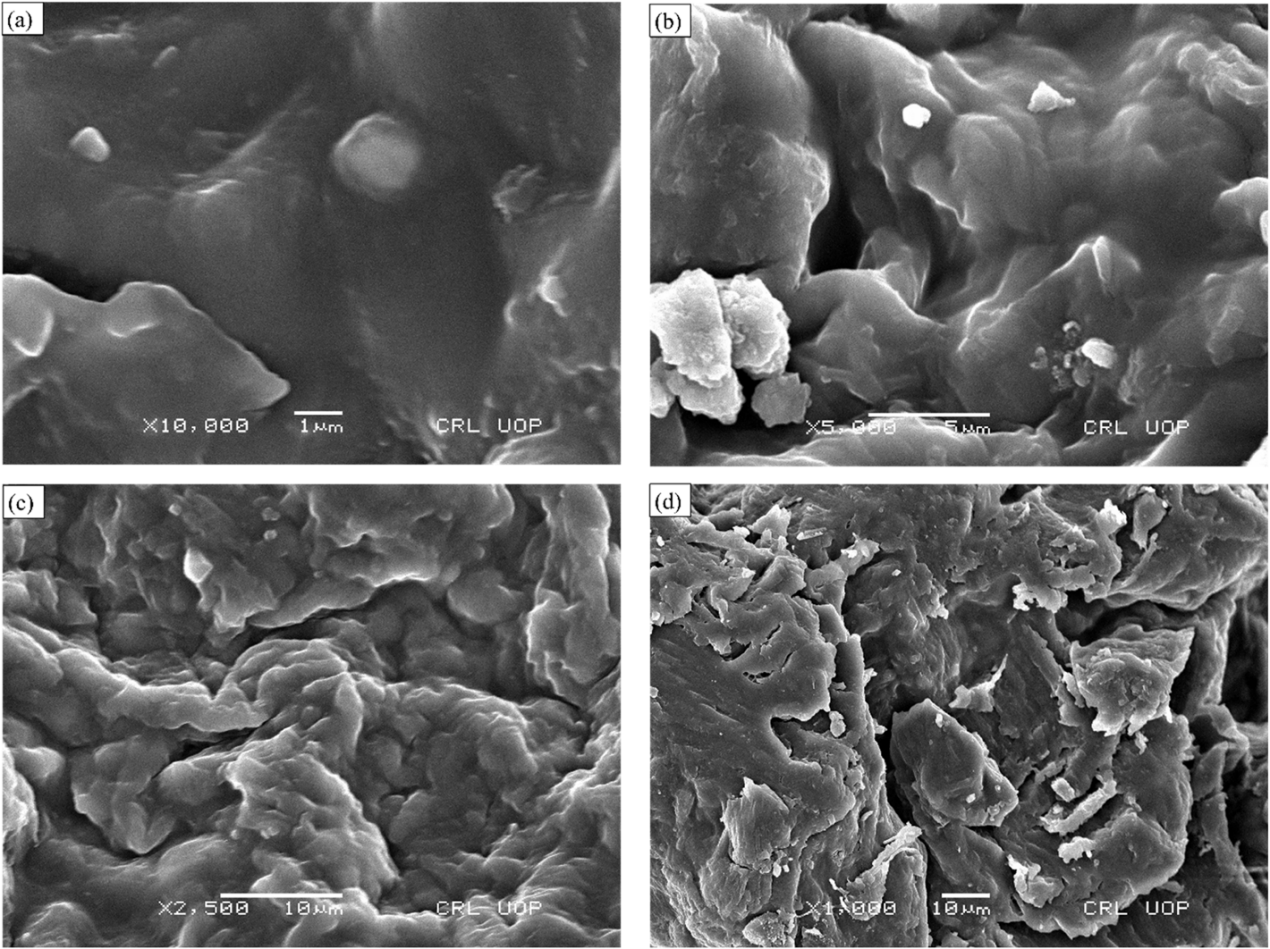
SEM analysis of CSGI hydrogel at (a) 1 μm and ×10 000, (b) 5 μm and ×5000, (c) 10 μm ×2500, (d) 10 μm ×1000.

SEM was used to determine the size, shape, and surface morphology of insulin-loaded chitosan hydrogel, sodium alginate, and graphene oxide. The SEM images of CSGI at high and low magnification are shown in Fig. 10. They reveal the spherical, or irregularly shaped sheet-like structure with highly porous, rough and smooth surfaces significantly enhanced by incorporating graphene oxide. The range at which they are magnified at high and low magnification is from 1 μm to 10 μm and depends on crosslinking conditions and formulation. The particle size was 600 nm as determined by Image J software. Adding graphene oxide increases porosity and mechanical strength, contributing to improved drug loading and release properties. The SEM analysis of CSGI with enlarged pores due to the large hydrophilic polar group increases hydrogen bonding, resulting in intercalation, swelling, and ion exchange, which leads in insulin release. These morphological characteristics highlight the suitability of CSGI hydrogel for sustained and controlled-release insulin delivery applications.^65–67^

### 3.7. Energy dispersive X-ray analysis

**Fig. 11:**
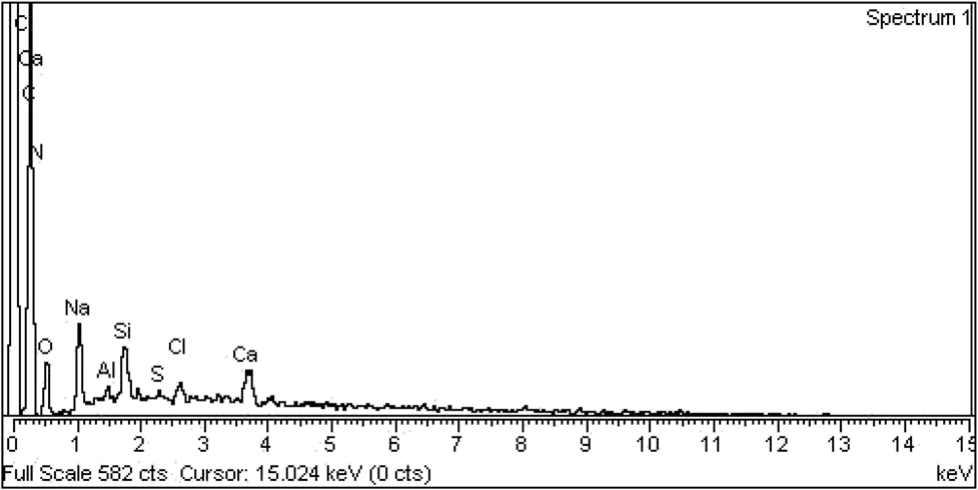
EDX spectrum of CSGI hydrogel.

Energy dispersive X-ray analysis is a key method to evaluate the elemental composition of CSGI hydrogel as shown in Fig. 11. The EDX spectrum commonly shows the presence of carbon (C), oxygen (O), nitrogen (N), Na, and silicon from TEOS, confirming the successful crosslinking of chitosan and sodium alginate polymer illustrated in the Table 1. The higher carbon-to-oxygen ratio reflects the incorporation of graphene oxide, while sulphur indicates the loading of insulin, and Cl, Ca are due to alginic acid and Al due to equipment contamination. Elemental mapping ensures the uniform distribution of graphene oxide, TEOS, and other components within the hydrogel matrix, ensuring structural homogeneity. The EDX analysis offers a comprehensive validation of the hydrogel composition, crosslinking efficacy, and suitability for controlled insulin release.^68,69^

**Table 1:** EDX of CSGI hydrogel weight and atomic percentage

Element	Weight %	Atomic %
C K	63.26	71.30
N K	9.22	8.91
O K	17.85	15.10
Na K	4.14	2.44
Al K	0.48	0.24
Si K	1.68	0.81
S K	0.23	0.10
Cl K	0.89	0.34
Ca K	2.24	0.76
Total	100.0	

### 3.8. Antimicrobial studies analysis

**Fig. 12:**
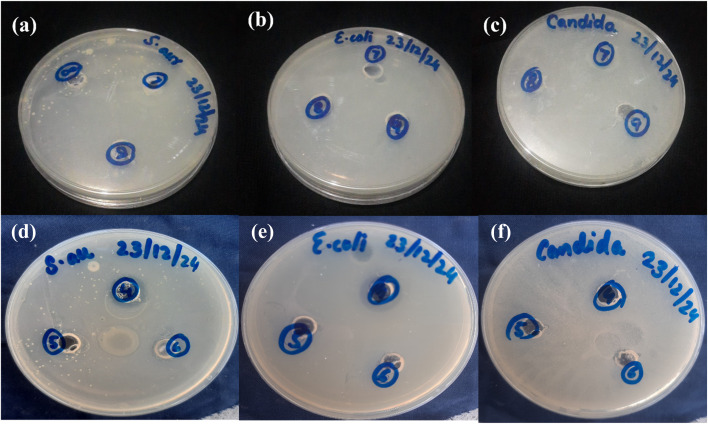
Antimicrobial activities of CSGI (7) hydrogel samples against: (a) *Staphylococcus aureus* (b) *Escherichia coli* (c) *Candida albicans*. Antimicrobial activities of CSG (6) hydrogel samples against: (d) *Staphylococcus aureus* (e) *Escherichia coli*, (f) *Candida albicans*.

The antimicrobial activities of insulin-loaded CSGI (7) and CSG (6) hydrogel without insulin were investigated against the following microbes: *Staphylococcus aureus*, *Escherichia coli*, and *Candida albicans* using a well-diffusion method. The diameter of the inhibition area of the zone of both bacteria and fungi is represented in Table 2. The observed inhibition zone indicated the release of insulin from the hydrogel and demonstrated the potential toxicity against bacteria, highlighting broad-spectrum antimicrobial activity, as shown in Fig. 12.^37,45^

**Table 2:** Antimicrobial activities of CSGI and CSG hydrogel

Test organisms	Zone of inhibition in mm		
	CSGI hydrogel	CSG hydrogel	Standard
*Staphylococcus aureus*	21.0 ± 0.4	0 mm	10 mm
*Escherichia coli*	10.0 ± 0.2	0 mm	27 mm
*Candida albican*	13.0 ± 0.3	0 mm	10 mm

### 3.9. Swelling study and its kinetics

**Fig. 13:**
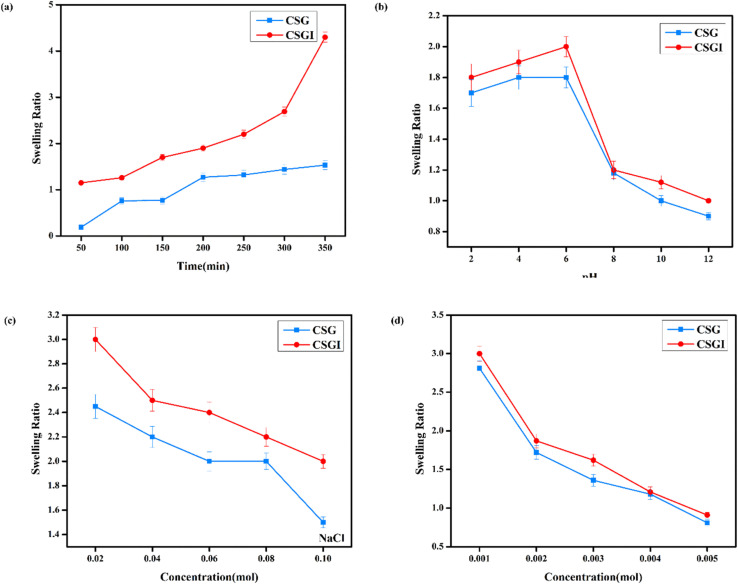
(a) Hydrogel CSG and CSGI swelling studies at the time. (b) Hydrogel swelling studies at different pH values. (c) Hydrogel swelling studies at different concentrations of NaCl. (d) Hydrogel swelling studies at different concentrations of CaCl_2_.

The swelling studies of CSG unloaded and loaded CSGI hydrogel were compared to determine how the drug affects water absorption capacity, mechanical strength, and stability. The sample CSGI loaded with insulin alters the swelling and equilibrium rates to check the kinetics of drug release. The swelling ratio of both samples was measured over 50 to 350 minutes, as shown in Fig. 13(a) at pH 7, and its swelling ratio consistently increased with time. However, the swelling ratio decreased when pH increased from 2 to 12, and this suggested that in acidic conditions, hydrogel shows more swelling than in an alkaline medium due to chitosan as it is a cationic polymer, it is protonated in acidic conditions due to electrostatic interaction between polymer chains. Alginate has more –COOH groups that deprotonate to –COO^−^ at elevated pH levels by increasing electrostatic repulsion in the hydrogel matrix, while chitosan is protonated from –NH_2_ to –NH_3_^+^.This dual ionization mechanism creates a finely tuned pH-sensitive system ideal for specific delivery of insulin, particularly in the intestine, where its absorption is favourable, as shown in Fig. 13(b).^70^ Also, the swelling ratio in different concentrations of NaCl and CaCl2. Ca^+^ can cause a more significant reduction than Na^+^ due to its divalent nature. This swelling results in network compaction, reducing free volume for water uptake. Higher salt concentration lowers the osmotic imbalance, which also reduces the swelling capacity, as shown in Fig. 13(c) and (d); it decreases with increasing salt concentration.^71,72^

The obtained loaded CSGI and unloaded CSG hydrogel swelling amount is determined by kinetic models first order, second order, and Korsmeyer Peppas models. The first-order kinetic is shown in eqn (6).

6 *M*_*t*_ = *M*_e_(1 − e^−*k*_w_*t*^)

Where *M*_t_ and *M*_e_ are the swelling amount at the time and maximum swelling amount at the time, *k*_w_ indicates the rate constant, and *t* is the time duration in minutes. The *k*_w_ value was calculated by linear fitting plots of −ln(1 − *M*_t_/*M*_e_) *versus t*, and the value of *R*^2^ is close to 0.94. It follows first-order kinetics, as shown in Fig. 14.7
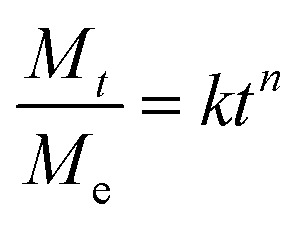
8
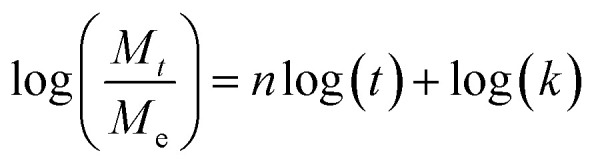


**Fig. 14:**
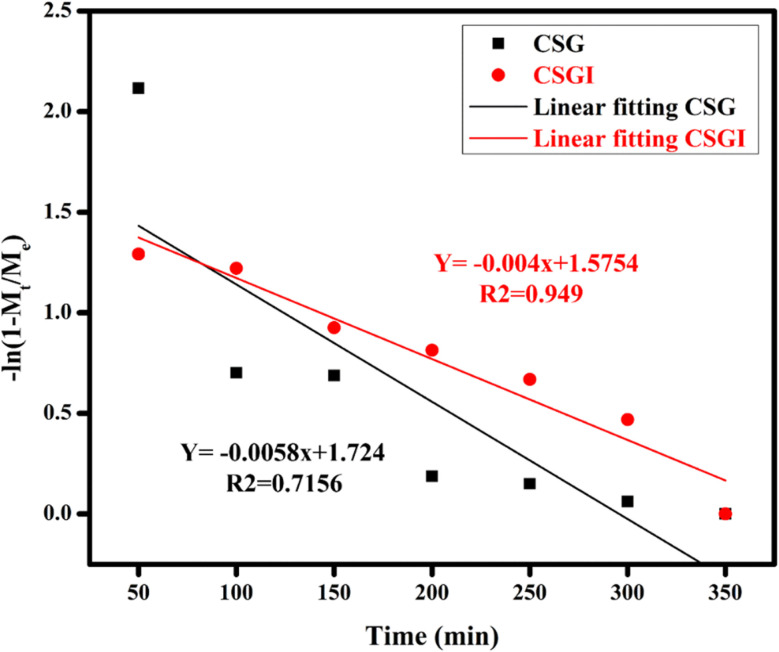
Swelling kinetics of first-order kinetic model by applying linear regression on hydrogel CSG and CSGI.

The second-order kinetics was determined by following eqn (9).9
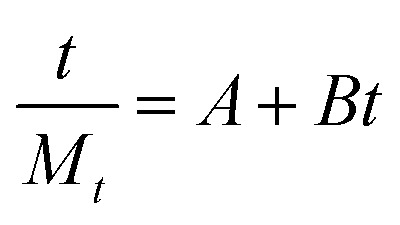
where *M*_*t*_ is the swelling amount at a time, *A* and *B* are constants related to initial and maximum swelling, and *k*_s_ is the second-order swelling rate constant, and it can be calculated by eqn (10) as shown in Fig. 15.10
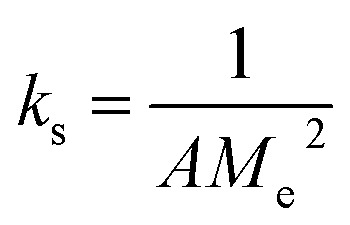


**Fig. 15:**
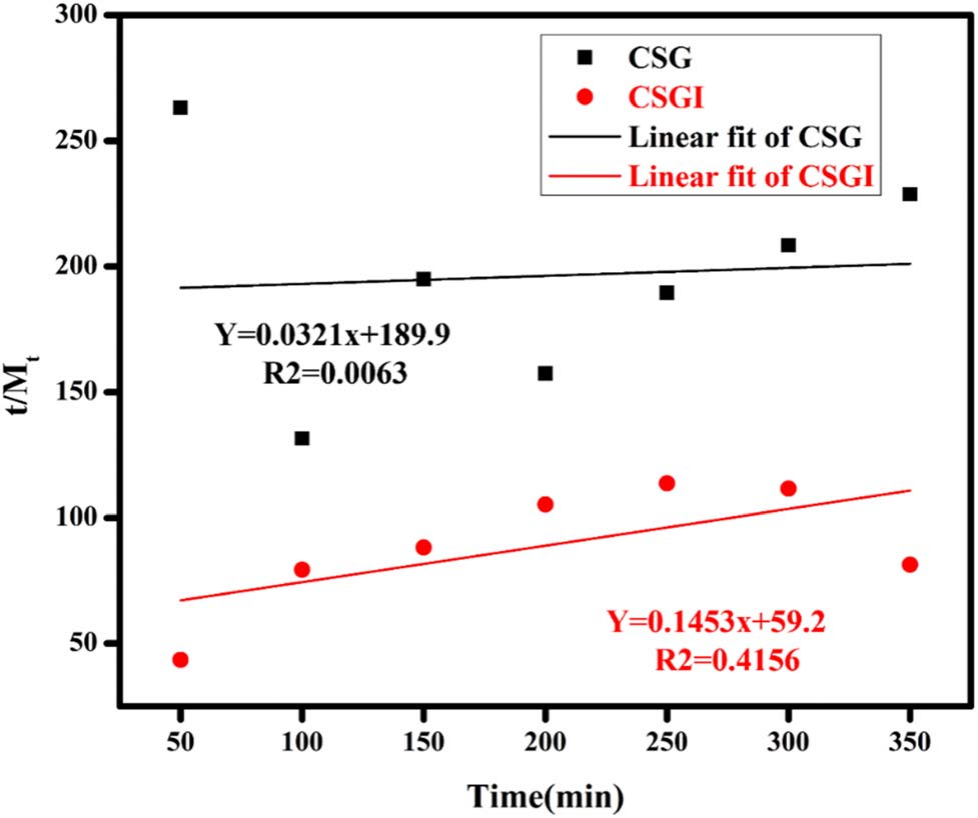
Swelling second-order kinetics of loaded hydrogel CSG and CSGI.

The Korsmeyer-Peppas kinetic model was applied, and the value of *n* diffusion exponent can be determined by eqn (11) as shown in Fig. 16.11
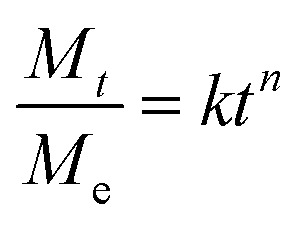
where *M*_*t*_ and *M*_e_ are swelling ratio at time and equilibrium or maximum swelling, *t* is time in minutes, diffusion exponent, *k* is rate constant, and eqn (12) can be linearized as12
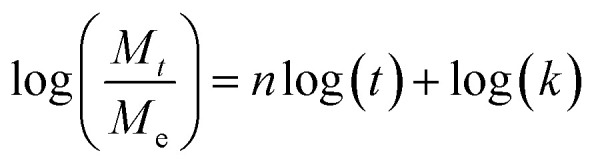


**Fig. 16:**
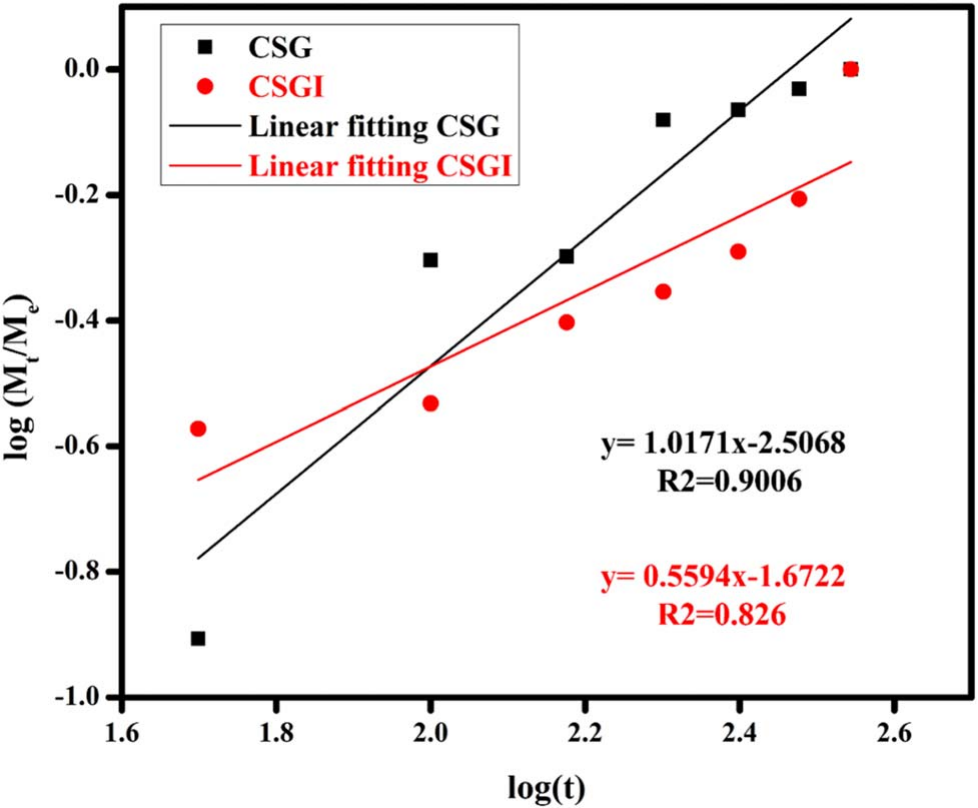
Swelling kinetics of Korsmeyer Peppas kinetic model of CSG and CSGI.

If the slope is less than *n* = 0.5, then it indicates Fickian diffusion, and our swelling exponent is *n* = 0.5 and 1 for CSG. It indicates Fickian diffusion, also, *R*^2^ is close to 0.826 for CSGI and 0.9 for CSG. It follows the Korsmeyer Peppas model; hence, swelling and drug release are primarily controlled by diffusion.^39,73^

### 3.10. Gel fraction analysis

**Fig. 17:**
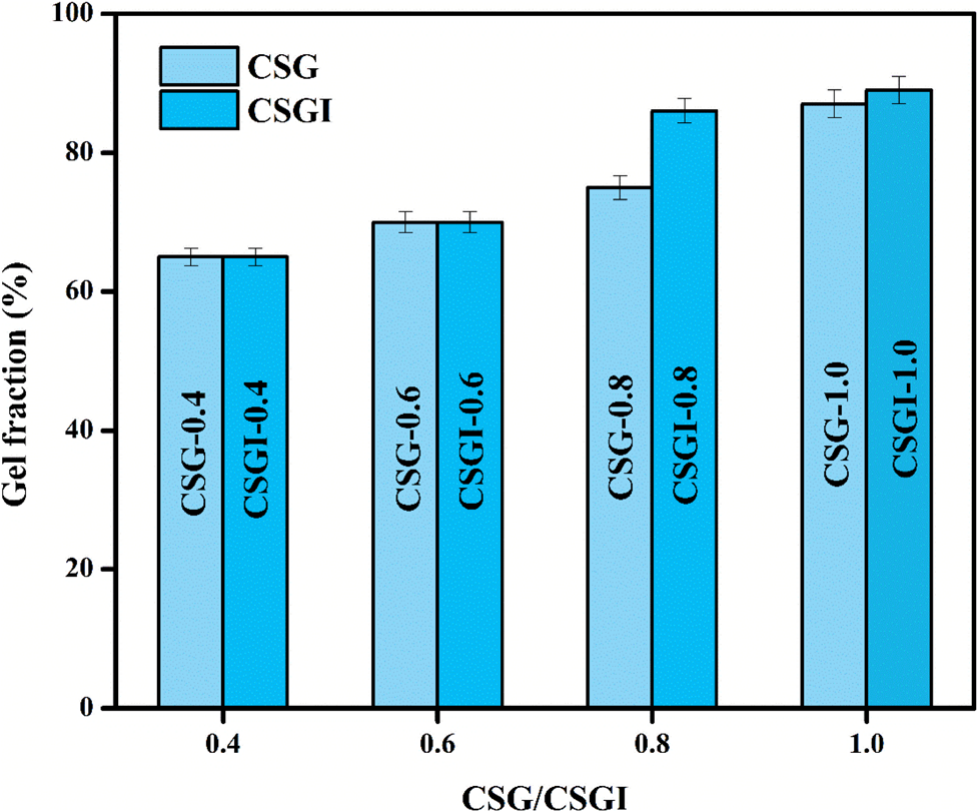
Comparison of gel fraction analysis for hydrogel samples CSG and CSGI.

The gel fraction analysis of hydrogel samples without insulin CSG and loaded with insulin CSGI, with the highest concentration of 1.0 g, are 86% and 89%. The increased gel fraction with insulin is due to increased crosslinking of TEOS, and with different concentrations of hydrogels is represented in a bar chart in Fig. 17. In this gel fraction, improvement is due to more crosslinking sites on chitosan and sodium alginate, which promote polymerization.^74^ A highly crosslinked hydrogel minimizes the leaching of unreacted monomers, which improves biocompatibility as loosely bound polymer chains provoke cytotoxic response. It may lead to a rigid network limiting cell adhesion. Thus, maintaining the gel fraction at its ideal level is crucial to striking a balance between cellular compatibility, degradability, and mechanical strength.^75^

### 3.11. Erosion studies

Erosion studies of hydrogel were conducted on different pHs from 2 to 12. The obtained results are given in Table 3 below:

**Table 3:** Erosion (%) of two hydrogel CSG and CSGI

Hydrogel	Erosion (%)	Hydrogel	Erosion (%)
CSG-2	72.4 ± 0.26	CSGI-2	69.5 ± 0.061
CSG-4	70.0 ± 0.01	CSGI-4	70.3 ± 0.033
CSG-6	67.7 ± 0.04	CSGI-6	66.6 ± 0.067
CSG-8	72.7 ± 0.02	CSGI-8	69.6 ± 0.01
CSG-10	73.3 ± 0.033	CSGI-10	66.0 ± 0.081
CSG-12	66.5 ± 0.01	CSGI-12	57.1 ± 0.014

The erosion behavior of chitosan, sodium alginate, GO, and insulin-loaded hydrogel CSGI exhibited some differences influenced by composition and other environmental conditions. Maximum erosion of CSG-10 was 73.3 ± 0.033, and for CSGI-8 was 69.6 ± 0.01 at pH 10 and 8 due to greater swelling at this pH. Also, insulin-loaded hydrogels displayed slightly reduced erosion compared to their non-loaded counterparts, which is due to increased crosslinking stability imparted by TEOS.^76–78^

### 3.12. Encapsulation efficacy (EE)

The EE of CSGI hydrogel was 94%, indicating highly effective drug loading. This high EE suggested an electrostatic interaction within the hydrogel polymeric matrix and insulin molecules. Also, the presence of graphene oxide enhances the retention of insulin molecules within the hydrogel. Such a higher EE indicates that CSGI is an excellent drug carrier for therapeutic drugs by ensuring minimal loss of drug and maximizing the dosage to be delivered at a targeted site.^79,80^

### 3.13. Post-encapsulation of insulin stability over time

**Fig. 18:**
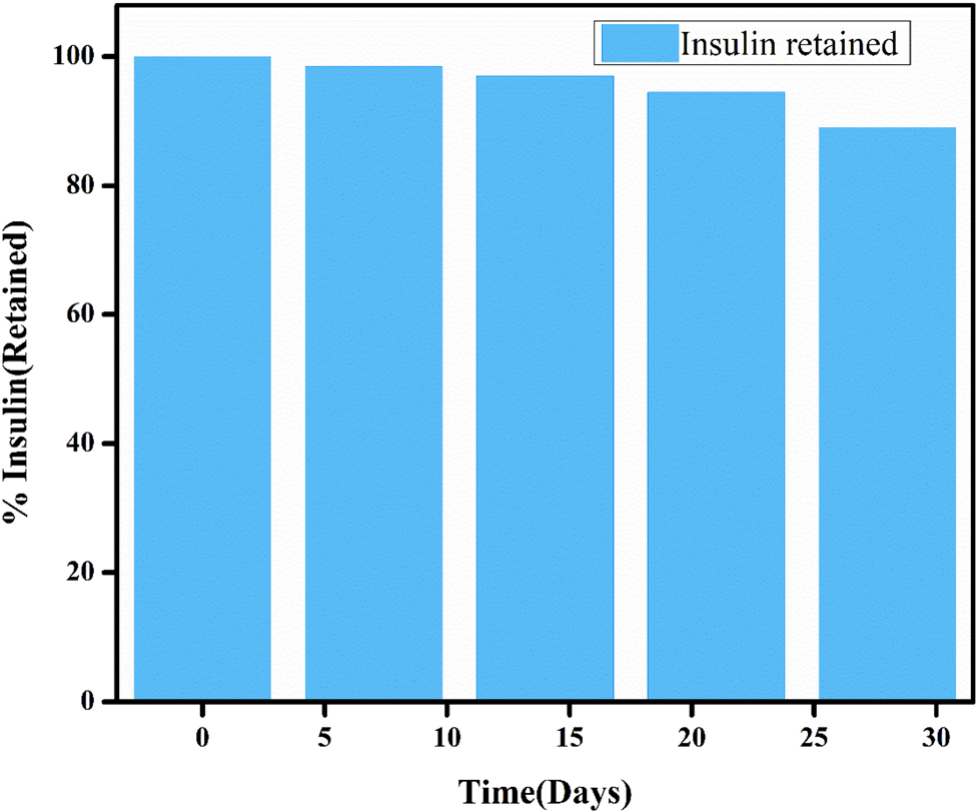
Post encapsulation assay represented in bars for insulin stability.

The results of this assay indicated that insulin-loaded hydrogel retained approximately 89% of its initial insulin content after 28 days of storage, which demonstrated the stability of encapsulated protein (Fig. 18). The slight decline indicated minor degradation; however, the retention of >90% insulin up to 21 days reflects the stabilizing effect of the CSGI hydrogel matrix. This protective environment is provided by chitosan and alginate polymers, which confirms that CSGI hydrogel is an effective and stable carrier for insulin delivery.^81,82^

### 3.14. Preparation of calibration curve equation

**Fig. 19:**
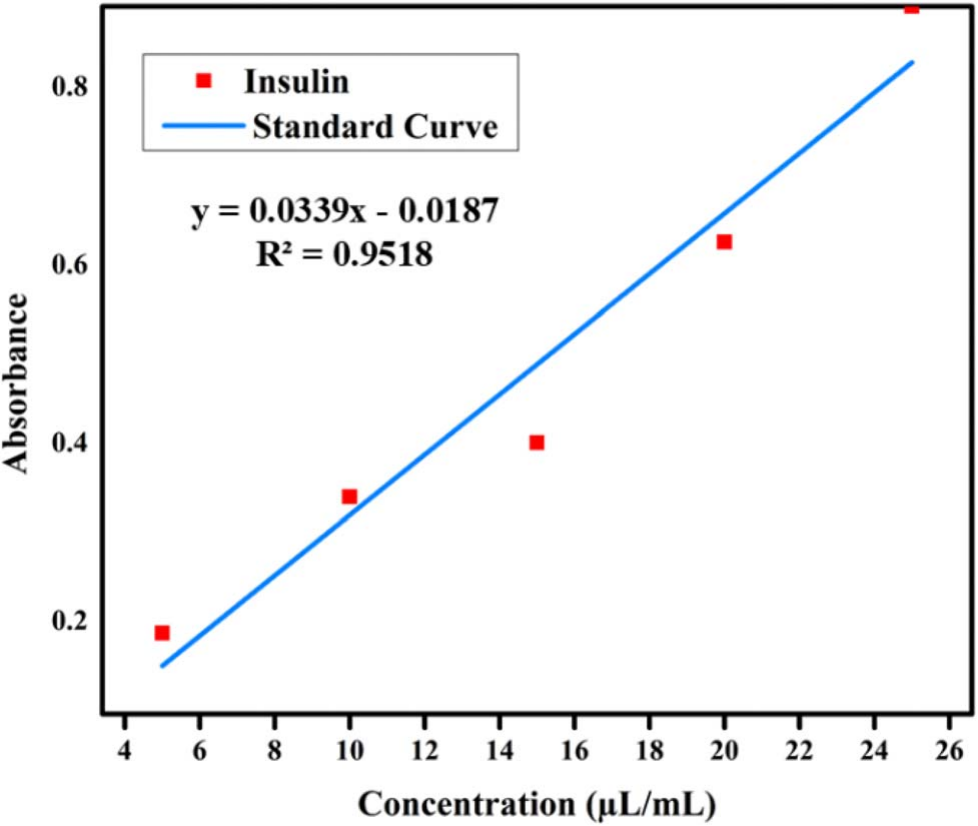
Standard curve for insulin.

A standard calibration curve was generated using a serial dilution from 5 μL to 25 μL in a 1 ml solution with pH 7. The resulting calibration curve demonstrated the linear relationship between insulin concentration and absorbance as described by a regression equation *y* = 0.0339*x* − 0.0187 with a coefficient of determination *R*^2^ = 0.9518 as shown in Fig. 19.

### 3.15. *In vitro* release studies

In vitro-release studies of hydrogel with chitosan, sodium alginate, and graphene oxide loaded with insulin (CSGI) samples were performed at three different pH levels and in a saline solution. To check the hydrogel's release profile, CSGI was monitored at pH 1.2, 6.8, and 7.4 with four different concentrations (0.4, 0.6, 0.8, and 1.0). The hydrogel sample CSGI was placed in a shaking incubating bath at 37 °C, and the sample was withdrawn at distinct periods, including 10, 20, 30, 45, 60, 90, and 120 minutes, respectively. After that, the sample was collected and diluted, and ultraviolet (UV) analysis was done to determine the concentration of insulin from the absorbance, which is at *λ*_max_ for insulin is 276 nm, from the data on insulin concentration. Drug release was calculated.^25,83^

**Fig. 20:**
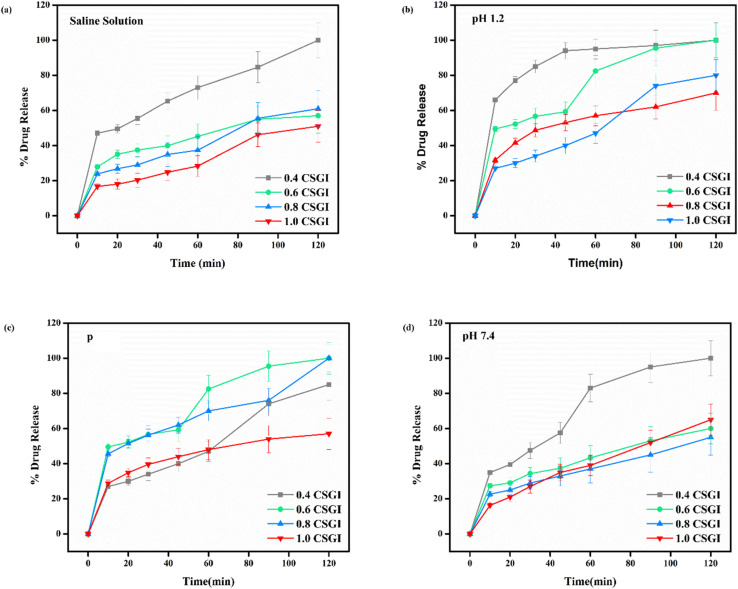
(a) % release of drug in saline solution, (b) % release of drug at pH 1.2, (c) % release of drug at pH 6.8, (d) % release of drug at pH 7.4.

From Fig. 20(a) in saline solution, the 0.4 g CSGI hydrogel demonstrated the highest insulin release rate, which is 80% within 120 minutes. Due to lower hydrogel concentration, the silica network, due to the crosslinker network, is more porous, and insulin can easily diffuse. As the concentration of hydrogel increases by 0.6, 0.8, and 1.0, insulin release decreases correspondingly because the hydrogel matrix is more crosslinked and the network is tighter. In Fig. 20(b) at pH 1.2 in gastric, the 0.4 g CSGI hydrogel has shown the lowest release of insulin, which is 80% in 120 minutes. This burst release was due to chitosan, which weakens its interaction in an acidic environment and results in a more rapid release of insulin. As the concentration of CSGI increases to 0.6, 0.8, and 1.0 g, insulin release is more gradual because of a denser network that limits diffusion even in an acidic environment, promoting chitosan dissolution. Also, in Fig. 20(c), pH 6.8 in the small intestine, the more sustained release was observed at 0.4, 0.6, and 0.8 g CSGI concentration due to chitosan being stable in the basic environment. Sodium alginate played a crucial role in regulating the release rate as it formed a network with chitosan that ionically crosslinked, preventing insulin diffusion. This alginate is particularly useful in ensuring that insulin is released at a more gradual and controlled rate in the small intestine. In Fig. 20(d) at pH 7.4, similar to blood pH, 0.4 g CSGI, the insulin release rate was higher. It followed the same trend as saline solution; as the concentration increased, the release rate decreased as the network that crosslinked was denser. So, overall results from the release rate suggest that insulin was released at a lower pH of 1.2 at a slower rate due to positively charged chitosan, but more sustained and controlled at pH 6.8.^84–87^ Its literature comparison is given in Table 4.

**Table 4:** Literature comparison for insulin delivery

Material	pH conditions	Release behavior	Stability	References
OCMC/SA-BSA nanohydrogel	pH 1.2, 6.8, 7.4	- pH 1.2: ∼36% after 3 h	Simulated oral digestion pH; stability studied in varying pH	59
		- pH 6.8: ∼50% after 48 h		
		- pH 7.4: ∼68.7% after 48 h		
3D-printed alginate–chitosan (Alg–Cs) scaffold	Neutral pH	Burst at 6–7 h	Designed for sustained insulin delivery, good retention over days	88
		- Alg75–Cs25/Ins10: ∼44.86% released in first 6 h		
		- Alg75–Cs25/Ins20: burst after 24 h, sustained up to 120 h		
SA/CS/SBC hydrogel + pancreatic β-cells	Not explicitly pH tested	Insulin secretion stable on days 1, 3, 5, 7	Focus on supporting β-cell activity, not just insulin passive release	88
CS/SA hydrogel crosslinked with TEOS, loaded with insulin	pH 1.2, 6.8, 7.4	(1) Saline solution: fast release	Stable under varying pH; suitable for oral administration simulations	My own work
		(2) pH 1.2 (gastric): burst release		
		(3) pH 6.8 (small intestine): sustained release		
		(4) pH 7.4 (blood): controlled release		

**Fig. 21:**
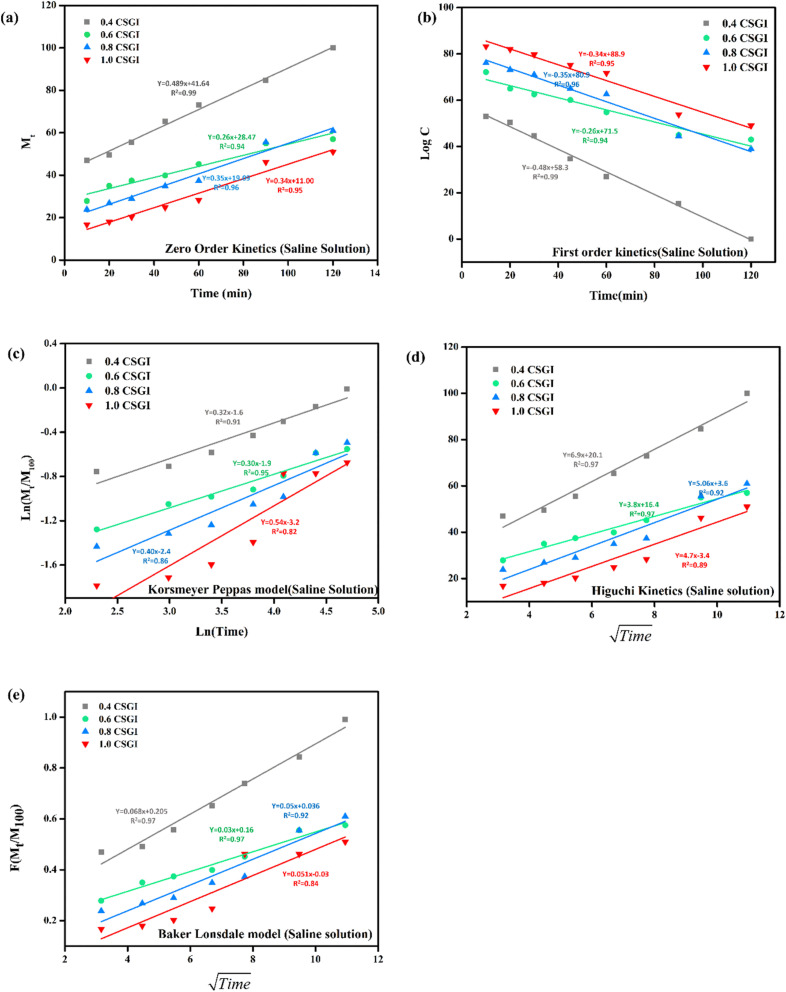
(a) Zero order kinetic model of saline solution. (b) First-order kinetic model of saline solution. (c) Korsmeyer Peppas model of saline solution (d) Higuchi kinetic model of saline solution. (e) Baker Lonsdale model of saline solution.

**Fig. 22:**
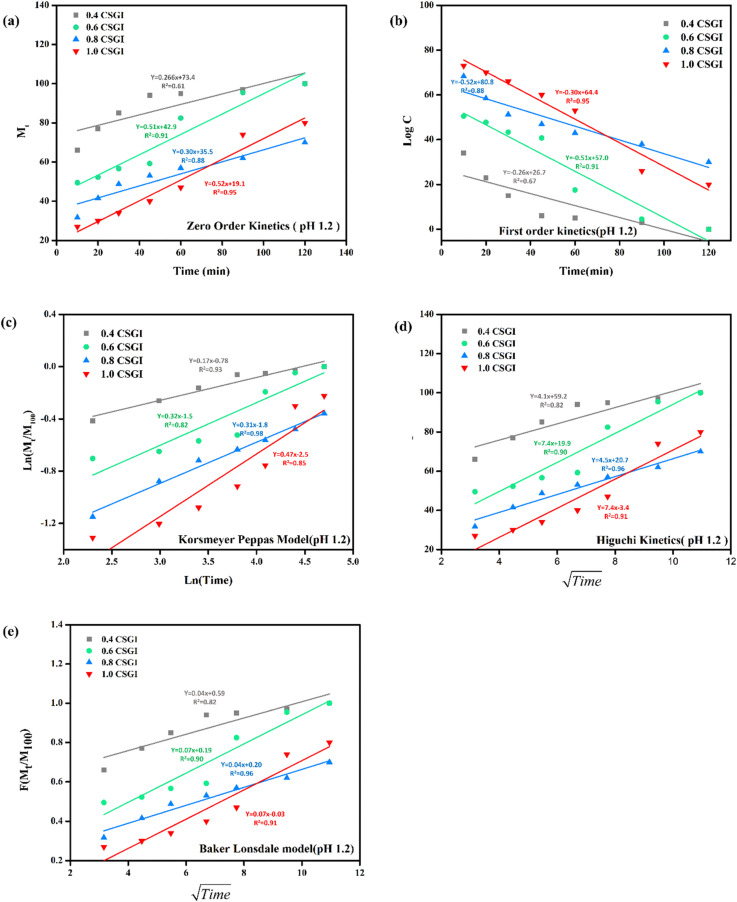
(a) Zero-order kinetic model at pH 1.2. (b) First-order kinetic model at pH 1.2. (c) Korsmeyer Peppas model at pH 1.2 (d), Higuchi kinetic model at pH 1.2. (e) Baker-Lonsdale model at pH 1.2.

**Fig. 23:**
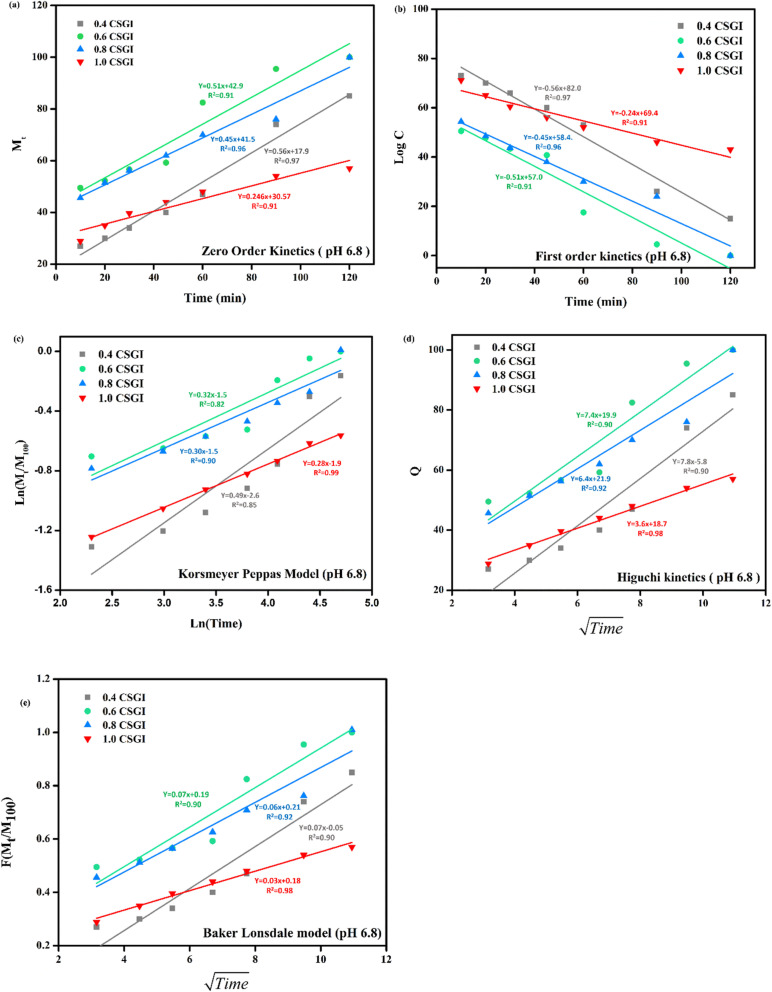
(a) Zero-order kinetic model at pH 6.8. (b) First-order kinetic model at pH 6.8. (c) Korsmeyer Peppas model at pH 6.8. (d) Higuchi kinetic model at pH 6.8. (e) Baker-Lonsdale model at pH 6.8.

**Fig. 24:**
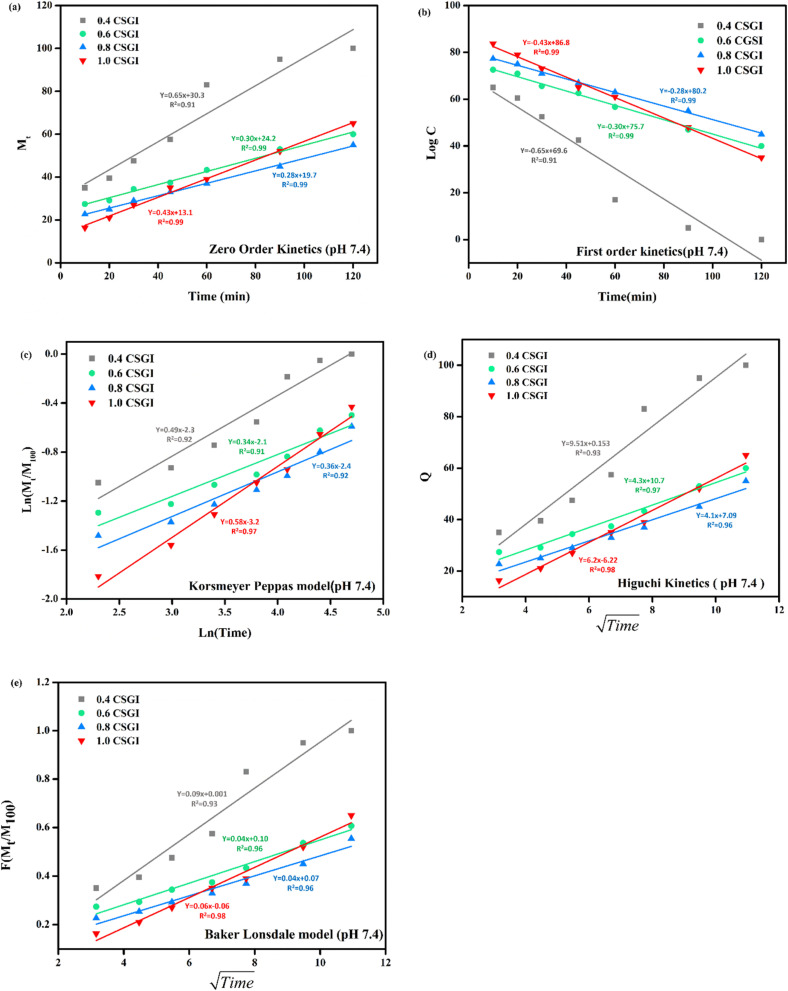
(a) Zero-order kinetic model at pH 7.4. (b) First-order kinetic model at pH 7.4. (c) Korsmeyer Peppas model at pH 7.4. (d) Higuchi kinetic model at pH 7.4. (e) Baker-Lonsdale model at pH 7.4.

The kinetics of CSGI hydrogel released the drug were applied to all conditions in saline and in three pH conditions as shown in Fig. 21–24, respectively. Then, from this data, we further checked where the release rate was higher, calculated its rate constant, and found out *R*^2^ values.

The zero-order kinetic model is shown in eqn (13)

13 *M*_*t*_ = *M*_∞_ + *K*_0_*t*

Where *M*_*t*_ is the release of the drug at the time, *M*_∞_ is the maximum release of drug, *K*_0_ is the rate constant, and the graph is plotted between the % drug release and time in linear fitting analysis. The first-order kinetic model is represented in eqn (14).14
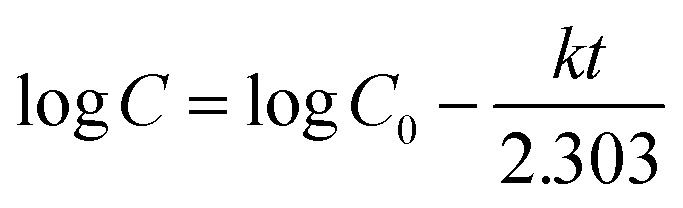
where *k* is the rate constant and time is in minutes, the graph is plotted between log *C*, the concentration of the remaining drug, and time in minutes. The Higuchi model is given in eqn (15).

15 *ft* = *Q* = *K*_H_ × *t*^1/2^

where *K*_H_ is the rate constant, *t* is the time, the graph is plotted between *t*^1/2^, and *Q* is the % drug release. The Korsmeyer Peppas model is shown by eqn (16).16
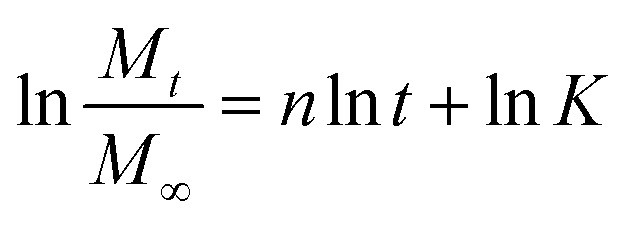
where *M*_*t*_ is the drug release at the time, *M*_∞_ is the maximum drug release, *K* is the rate constant, and the graph is the plot of natural log time and natural log % drug release. Baker Lonsdale kinetic model is represented by eqn (17).17
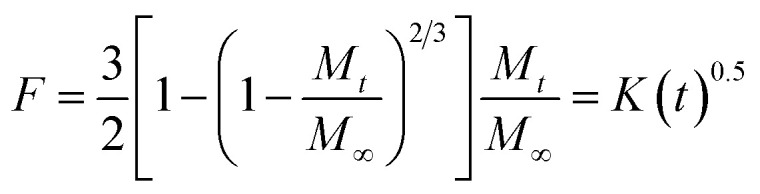
where the graph is plotted between *t*^0.5^ and *F* is the fraction released which is *M*_*t*_/*M*_100_ where *M*_*t*_ is the % release of drug at a specific time and *M*_100_ drug released at a maximum time which is 100%.^89–91^

From this study, as shown in Fig. 21–24. It was further confirmed by kinetic models that maximum insulin release was at pH 6.8 of CSGI hydrogel, whose rate constant values and regression coefficient *R*^2^ values are shown in Table 5. The constants *K*, *K*_0_, and *K*_H_ in the formula are characteristic constants. The kinetic analysis demonstrates that a slower and more sustained release rate was observed at 1.0 g CSGI, and the regression coefficient and rate constant confirmed it. The *R*^2^ values for zero order, first order Korsmeyer Peppas model, Higuchi model, and Baker Lonsdale model are 0.99, 0.97, 0.99, 0.98, and 0.98, respectively. These higher *R*^2^ values and constant *K*_0_ 0.56 and *K*_H_ 7.8 suggest the controlled diffusion, and *n* = 0.49 indicates that it follows the Fickian diffusion mechanism. Overall, these results indicated that 1.0 g CSGI more sustained and controlled insulin release, making it optimal for therapeutic application.^92–95^

**Table 5:** Comparison of kinetic model by *R*^2^ values

Conc. (g)	Zero-order *R*^2^	Zero order *K*_0_	First order *R*^2^	First order *K*	Korsmeyer Peppas *R*^2^	Korsmeyer Peppas *n*	Higuchi model *R*^2^	Higuchi model *K*_H_	Baker Lonsdale *R*^2^	Baker Lonsdale *K*
0.4	0.97	0.51	0.97	0.51	0.85	0.49	0.90	7.8	0.90	0.04
0.6	0.91	0.56	0.91	0.56	0.82	0.32	0.90	7.4	0.90	0.07
0.8	0.96	0.45	0.96	0.45	0.90	0.30	0.92	6.4	0.92	0.06
1.0	0.99	0.24	0.91	0.24	0.99	0.28	0.98	3.6	0.98	0.03

#### 3.15.1 Statistical analysis by ANOVA

**Fig. 25:**
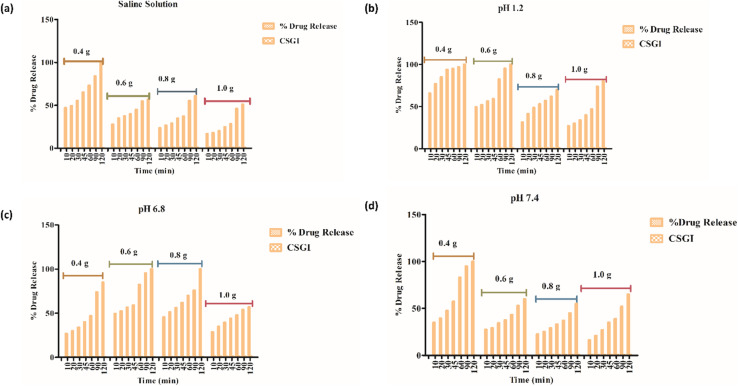
Two-way ANOVA on drug release studies by GraphPad Prism: (a) in Saline solution, (b) at pH 1.2, (c) at pH 6.8, (d) at pH 7.4.

Two-way ANOVA indicated that the column factor of hydrogel CSGI contributed 71.08% of the total variation and was statistically significant (*p* < 0.0001, ***). The row factor (time or replicate variation) was not significant (*p* = 0.5314, ns), contributing only 14.24% of the variation. This result demonstrated that the treatment has a stronger effect on the response variable in saline solution than on time-dependent variables in saline solution. With a highly significant (*p* < 0.0001 ***), the column factor explained 79.66% of the total variation, indicating a dominant treatment effect in acidic conditions. With a contribution of only 10.03% to the variation, the row factor was once more not significant (*p* = 0.5276). At pH 6.8, a similar pattern was observed, with the column factor accounting for 78.68% of the overall variation (*p* < 0.0001, ***), suggesting a significant treatment effect. The row factor only accounted for 10.64% of the variation and was still statistically non-significant (*p* = 0.5043). The column factor, which accounted for 68.97% of the variation, was still significant at pH 7.4 (*p* < 0.0001, ***). With an influence of 15.35%, the row factor once more failed to demonstrate significance (*p* = 0.5220). Treatment differences (*e.g.*, formulation or concentration) had a significant impact on drug release for all tested environments (saline and different pH levels). The type or concentration of the hydrogel formulation used is the primary determinant of release kinetics, even though drug release does vary over time, as time intervals did not significantly contribute to variation (Fig. 25).

## 4. Conclusion

Various formulations of hydrogel CSG and CSGI were successfully synthesized and thoroughly characterized using physicochemical and pharmacological techniques to assess their potential for controlled insulin delivery. SEM verified the homogenous dispersion of components within the hydrogel matrix, while EDX analysis confirmed the compositional purity of the synthesized material. XRD analysis demonstrated the amorphous nature of sodium alginate, as evidenced by its low-intensity peaks, where distinct peaks of chitosan and graphene oxide confirmed its crystalline structure. Swelling studies were conducted under varying conditions, including changing time and at different pH conditions, exposure to saline solution, and in CaCl_2_. Swelling studies revealed that the hydrogel exhibited maximum swelling at neutral pH and at pH 6.8, where polymer relaxation and water uptake were optimal. In acidic conditions at pH 2, swelling was lower due to the protonation of chitosan, which led to intermolecular interaction restricting water absorption. Conversely, at fundamental pH 12, swelling is decreased due to polymer deprotonation, resulting in network contradiction and reduced water retention. *In vitro*, the release was conducted at three specific pH levels (1.2, 6.8, 7.4) and in saline solution to mimic the physiological environment. Maximum release occurs at a pH buffer of 6.8, resembling a small intestine environment in which insulin is released controlled and sustained. So, the results described slower and sustained release in the basic environment, as proved from the above table of pH 6.8, where the regression coefficient and rate constant represented release but the degradation of protein insulin in harsh environments at pH 1.2. Overall, this research highlights the potential of CSGI hydrogel as a promising carrier for the sustained release of insulin, offering an effective strategy to enhance its therapeutic efficacy. Future investigations should focus on *in vivo* evaluations to validate the hydrogel's biocompatibility and pharmacokinetic profile, alongside stability assessments and formulation optimizations to enhance its clinical applicability for diabetes management.

## Conflicts of interest

There are no conflicts to declare.

## Data Availability

The data supporting the findings of this study are available from the corresponding author upon reasonable request.
